# A tunable acoustic absorber using reconfigurable dielectric elastomer actuated petals

**DOI:** 10.1038/s44172-023-00159-z

**Published:** 2024-01-10

**Authors:** M. Shrestha, G. K. Lau, Y. W. Chin, E. H. T. Teo, B. C. Khoo, Z. B. Lu

**Affiliations:** 1 Continental-NTU Corporate Lab, Nanyang Technological University, Singapore, 639798 Singapore; 2 National University of Singapore, Singapore, 117411 Singapore; 3 Department of Mechanical Engineering, National Yang Ming Chiao Tung University, Hsinchu, 300093 Taiwan; 4 School of Electrical and Electronic Engineering, Nanyang Technological University, Singapore, 639798 Singapore; 5 School of Materials Science and Engineering, Nanyang Technological University, Singapore, 639798 Singapore; 6 School of Aeronautics and Astronoautics, Sun Yat-Sen University, Shenzhen, 518107 PR China

**Keywords:** Mechanical engineering, Soft materials, Applied physics

## Abstract

Dielectric elastomer actuator (DEA)-based unimorphs that actively bend in one direction, can mimic the blooming motion of flower petals. Here we explore an application of such reconfigurable DEA to create tunable acoustic absorber capable of adapting to fluctuations in dominant noise frequency. The DEA-unimorphs consist of alternate layers of dielectric elastomers and compliant electrodes bonded to a Mylar sheet and were micro-slotted to form triangular petal-like structures that bend upon voltage activation. When arranged in an array, the micro-slotted dielectric elastomer bending actuators (MSDEBA) can open like flower petals, actively reconfiguring their open-ratio. Integrated with a base resonator comprising a micro-slotted panel (MSP) and a parallelly arranged varying-depth (VD) back-cavity, the MSDEBA forms a tunable acoustic absorber effective in the low-mid acoustic frequency range at inactive state. Meanwhile, upon voltage activation, it increased the absorber’s open-ratio and tuned the absorber to target a higher frequency. A 5 kV activation reconfigured the MSDEBA to shift its transmission loss peak by 72.74% (i.e., from 697 Hz to 1204 Hz). This acoustic spectrum tuning capability doubled the 15 dB absorption bandwidth of these absorbers from a bandwidth of ~435 Hz to 820 Hz. Such absorbers have the potential to tune the absorption spectrum to match the noise frequency in real-time to ensure optimal acoustic attenuation.

## All

The fixed and narrow working frequency bandwidth limits the use of most acoustic absorbers in a dynamic acoustic environment. Shrestha and colleagues designed a tunable acoustic absorber capable of absorbing sound with varying frequency bands using dielectric elastomer bending actuators. The triangular petal-like structure actively reconfigures its shape by voltage activation to shift the noisy absorption spectrum, offering a practical solution for real-time outdoor noise absorption.

## Introduction

Noise remains a great source of discomfort to many in dense cities with rapid redevelopment. While the thick wall of a house provides great noise insulation, openings for ventilation and views such as windows and doors allow entry of urban noises^1^. Traditionally, thick and porous absorbers such as foams and rockwools would work sufficiently to absorb noises inside the house, but they are opaque, and risk being soaked up if exposed to rainwater. In addition, urban noise sources like fans, machines, construction, mass transport and speech are concentrated in the lower but wide frequency range of 50-2500 Hz^2,3^, in comparison to human perceptible sound frequency range of 20-20000 Hz. In addition, high-frequency noises attenuate rapidly with distance travelled. Therefore, increasingly noise barriers that can absorb a wide range of low-frequency noises are becoming more desirable. However, until today, most outdoor acoustic absorbers are designed for a fixed and narrow acoustic frequency bandwidth, often only reducing noises around ±50 Hz range about a peak frequency^4^.

Recently advances in sound-absorbing materials, acoustic metamaterials along with coupled resonant systems have made it possible to achieve high-efficiency and relatively broadband sound absorption using thinner structures at deep-subwavelength scales^5,6^. Some were even designed for multifunctional situations like periodic resonators^7–9^, sonic crystals^10–12^, and resonant membrane absorbers were widely employed with the aim to make thin transparent absorbers^13–17^ for windows applications. Among them, membrane-type absorber absorbs the noise in the low to middle-frequency range, eliminating the need for very thick, porous absorbers. Special designs of resonant absorbers have also been developed to simultaneously absorb sound and allow air circulation for ventilation^18–21^. However, most of these absorbers still had a narrow absorption band tied to a fixed resonant frequency^22–24^. Tunable membrane absorbers were developed using a pre-stretched dielectric elastomer actuator (DEA)^25–28^. A high voltage activation of the DEA reduced the membrane tension (by applying the Maxwell stress^29^ and thus lowered the absorber’s resonant frequency)^26,30^. Despite this active adaptive property, such an absorber is ineffective for relatively broader real-world noise.

On the other hand, recently developed microperforated panel (MPP) and micro-slotted panels (MSP) absorbers^31–34^, obtain a broader band of sound absorption. Yet, its sound absorption capability is usually insufficient for a general-purpose absorber. For instance, common MPP with an orifice diameter in the range of 0.5–1.0 mm can achieve a half absorption (i.e., the sound absorption coefficient > 0.5) bandwidth of one to two octaves. Both the bandwidth and the half-absorption performance are lagging to compete with the porous absorbers. A tunable MPP or MSP based on reconfiguring the dimensional characteristics of these panels is an investigated solution. The MPP and MSP consist of a rigid panel with submillimeter perforations (holes) or slots and a back cavity. Its resonant frequency depends on the hole sizes (i.e., diameter and depth) and the back-cavity volume^31,35,36^. Studies have used screw adjustment^35^ or a stepper motor^37,38^ to change the back-cavity volume of the MPP absorber. These cavity tuning methods are however impractical and costly for large-area MPP. Recently, an electrically tunable acoustic absorber based on a micro-perforated dielectric elastomer actuator was demonstrated^39–42^. These devices have a broader band attenuation of low-to-medium frequency sound and in addition, are capable of electrically tuning the peak absorption frequency and bandwidth. This is achieved by voltage activation that reduces the membrane tension and thus hole size. However, these devices can only survive for a few months as the stretched elastomer membrane with holes ruptures due to the creep of the pre-tensioned viscoelastic elastomer in a short period of time, making them impractical for real applications.

Plants are natural sound absorbers, and they could inspire us to develop an absorber that meets the current need for acoustics attenuation. It is well known that dense tree leaves can absorb noises^43^, such that trees are planted as visually appealing landscapes along housing estates beside busy roads to isolate traffic noise. Besides leaves, soft flower petals are thought to provide sound absorption by local resonance at low frequencies and create band gaps in the sound transmission frequency spectrum^44^. Based on simulations of fully blossomed rose flowers, Chen et al.^44^ proposed that arrays of millimetre-sized flower-like acoustic metamaterial unit cells could increase band gaps by 3 times in the low-frequency region below 400 Hz, compared to unit cells without the petal. The unit cell consists of a 3.2 mm-radius hard tungsten hemisphere encircled by a 1.5mm-high, 0.5mm-thick petal-shaped silicone rubber and is supported by a silicone rubber back plate with a back cavity. They find that band gaps are increased by larger hemisphere radius and thicker but smaller back support plates. This suggests that the larger and thin petals can reduce noise in wider bandwidth. In addition, there are plants that can demonstrate nastic motion in response to external stimuli, like thigmonastic movements in Dionaea, Utricularia, Aldrovanda, Drosera and Mimosa^45^. Some flowers also respond to stimuli like light, temperature and endogenous rhythms by blooming and closing^46,47^.

**Fig. 1:**
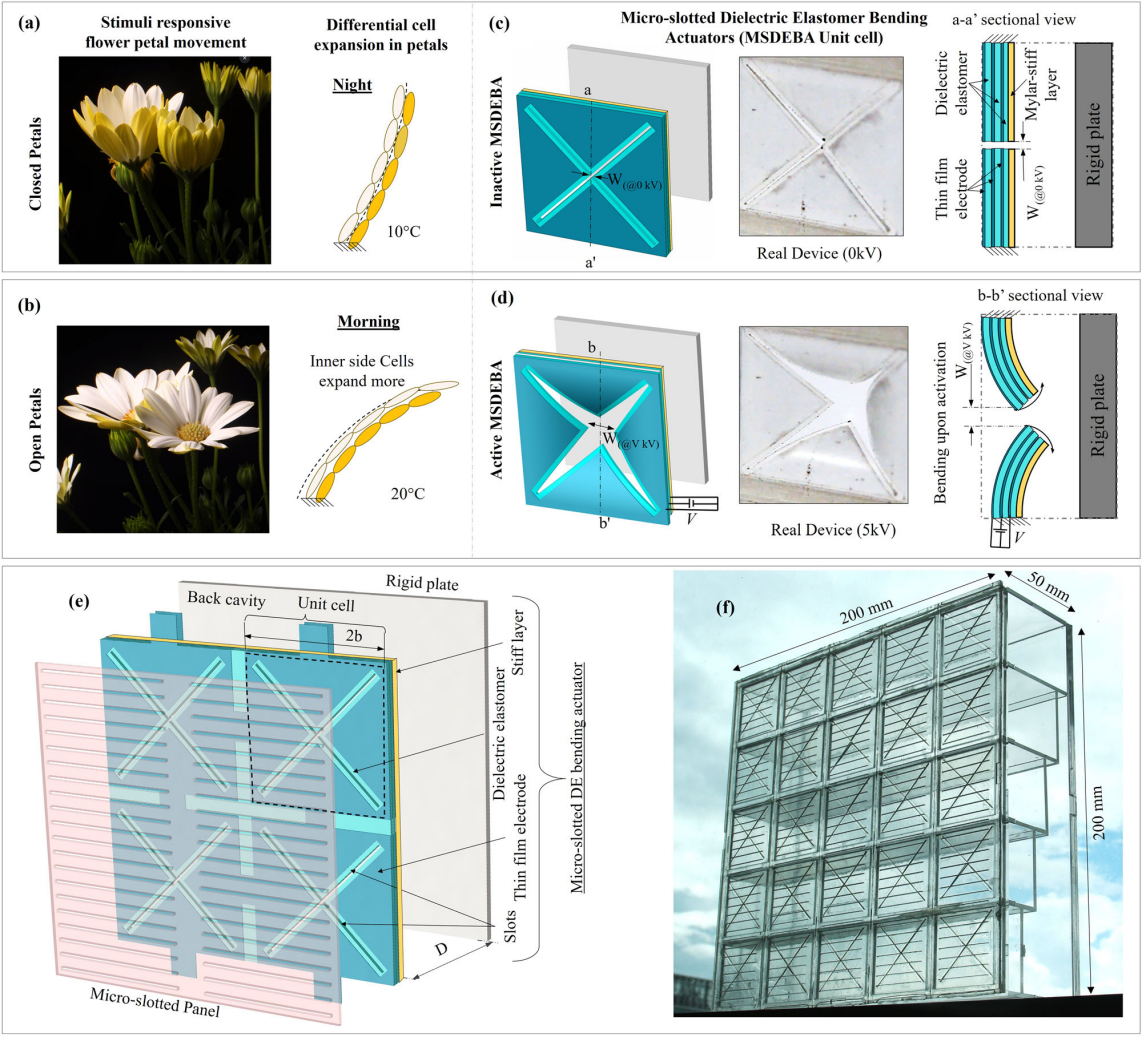
Bioinspiration and working principle of micro-slotted dielectric elastomer bending actuator (MSDEBA)-based transparent tunable acoustic absorber. **a** Opening and **b** closing of flower petals as a response to light or heat stimulus and a physics behind the petal’s bending motion; Analogous working principle of a MSDEBA which works like the petals through differential expansion of one side relative to the other, but the stimulus here is variation in sound frequency and is driven by high voltage. Isometric and side cross-sectional view of a unit cell of MSDEBA (**c**) at inactive state. **d** At activated states. **e** Exploded view of micro-slotted panel (MSP)/MSDEBA/Back-cavity components of the tunable acoustic absorber. **f** Photo of a real tunable acoustic absorber device.

This work seeks inspiration from the shape and blooming motion of flower petals to make a flowers-shaped acoustic absorber that can efficiently absorb sound and simultaneously adapt to variations in the frequency of sound by changing its shape. The petal-like features of the absorber can aid in absorbing sound and the ability to curl and uncurl petals like booming flowers allows it to target the desired frequency of sound. This petals-inspired acoustic absorber consists of an acoustic resonator made of a front micro-slotted panel (MSP) and a parallel-arranged variable-depth (VD) back-cavity with the add-on of biomimetic dielectric elastomer petals in between. The dielectric elastomer petals are made of a micro-slotted dielectric elastomer bending actuator (MSDEBA) whose individual petals work like a DEA unimorph^48,49^ and are the actively reconfigurable component that mimics the blooming motion of flower petals. The MSDEBA consists of a multi-layered dielectric elastomer actuator with one non-stretchable and flexible Polyethylene terephthalate (PET) layer making them a multilayered DEA unimorph^50–52^. As shown in Fig. 1, the opening of dielectric elastomer petals upon high voltage activation will expose a larger area of MSP whereas its closing will cover the MSP. This allows tuning of the effective hole or open ratio of the MSP and thus tunes the resonant frequency. The increase in open ratio due to voltage-induced bending of dielectric elastomer petals causes shifting of the acoustic absorption spectrum to a higher frequency. This voltage-controllable shifting of the absorption spectrum helps the absorber to adapt to variations in noise frequency in real time and ensures optimal absorption. Unlike micro-perforated dielectric elastomer actuators, in the MSDEBA the elastomer is stretch-free and bonded to stiff and non-stretchable PET membranes eliminating the possibility of creep and rupture. In addition, fabricating micro-slots and modifying the micro-slot dimensions to address different frequency ranges is much simpler and more scalable compared to micro-perforations.

## Results and discussion

### Petal-shape DEA opening with large bending

Most flowers open and close upon the right type of stimulation. Changes in light, temperature, humidity, or even endogenous rhythms trigger the blooming and closing of flowers. Flowers of the daisy family open when exposed to light and close with darkness (see Fig. 1a, b). In crocus or tulip, the increasing temperature at sunrise triggers the flower to open wide. As the daylight wanes and the temperature drops the petals close. Usually, petal movements are due to a difference in the expansion rate and/or growth of the two sides of the petals over a short or long period. When the inner surface of the petal rapidly grows or expands in length while the outer surface does not, it causes opening (see Fig. 1b). Similarly, when the more rapid growth of the outer surface occurs it causes closing of the flower (see Fig. 1a)^46,47^. Such differential expansion between bonded layers can cause the bending of large-area sheets. Inspired by petal movement, we have devised a multilayer MSDEBA system with each unit consisting of four petal-like segments. Each of these dielectric elastomer petals is a transparent piece of unimorph in a triangular shape. For simplicity of fabrication, overlapping between dielectric elastomer petals is avoided, and dielectric elastomer petals are separated by a micro-slit of 0.36 mm width using a 2D laser cutting machine. Each unit of MSDEBA is similar to a flower/pinecone and is a square of 40 mm in length, while the triangular-shaped quadrants are free and behave like petals^53–55^. Each triangle-shaped unimorph consists of multi-layered DEA resting on a flexible PET substrate. The multilayered DEA consists of three soft VHB layers each of 250 µm thickness and the PET is 25 µm thick. The multilayered DEAs with thinner elastomer layers were used instead of a single elastomer layer of the same total thickness to reduce the needed applied high-voltage for the DEA. The outer two VHB layer is sandwiched between the transparent PEDOT:PSS/AgNW electrode layers forming a transparent DEA. A high voltage activation of the dielectric elastomer petals results in areal expansion of the dielectric elastomer layers which is constrained by a non-stretchable PET layer. Consequently, like in petals, the expansion stress is converted to bending stress. This results in saddle bending with the PET surface as the concave side. Consequently, the saddle bending of triangular MSDEBA quadrants or dielectric elastomer petals leaves a larger gap between the adjacent quadrants mimicking an opening motion (see Fig. 1c, d). The front view and the side view videos of the MSDEBA’s opening motion are presented in Supplementary Video 1 and 2 respectively.

### Design and bending model of MSDEBA absorber

The MSDEBA absorber module is a box of 200 mm in length and 200 mm in breadth with a thickness of 50 mm. Figure 1e, f shows the acoustic absorber module which consists of a front micro-slotted panel (MSP), a second MSDEBA layer and a parallelly arranged varying depth back cavity. The 200 mm square MSP/MSDEBA layer faces the noise source. The MSP panel consists of a 1 mm thick acrylic plate with 400 micro-slots of 28.28 mm length and 0.35 mm width distributed uniformly with a 2.5 mm spacing. Each MSDEBA module consists of 25 unit-MSDEBAs, arranged in five rows and columns. Figure 1c shows a unit-MSDEBA that has a diagonally cut micro-slot of 42 mm lengths. It is assembled to be in direct contact with the MSP with the outer facing mylar film side. At the inactive state, the overlapping MSDEBA blocks the micro-slot of the MSP except for the central overlapping holes. The bending of the MSDEBA quadrants or the dielectric elastomer petals upon high voltage activation (refer to Supplementary Video 3) increases the open ratio of the MSP (refer to Supplementary Video 1) and alters the acoustic properties of this absorber.

**Fig. 2:**
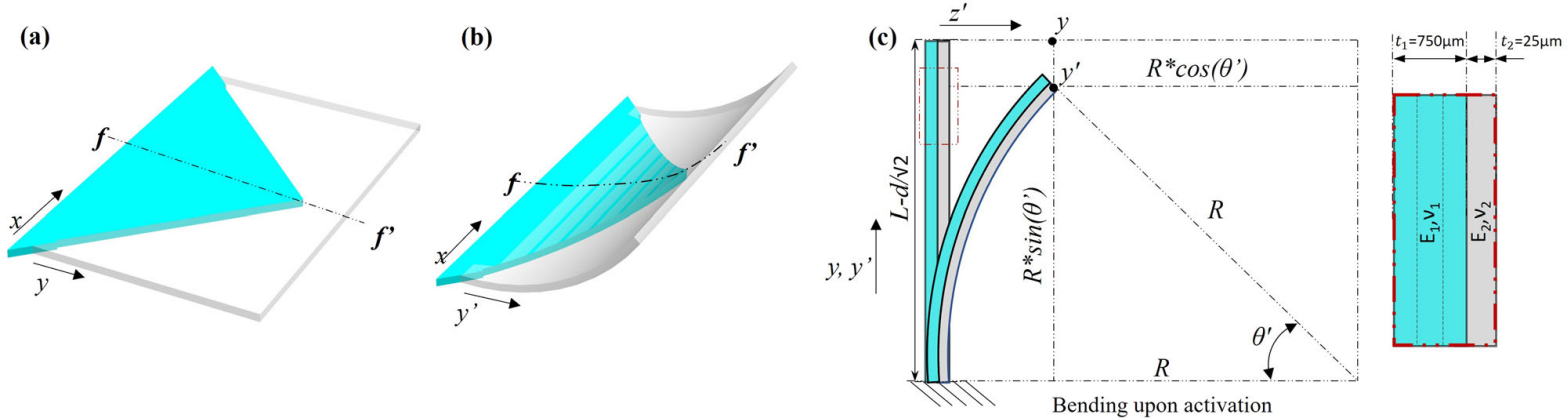
Electromechanical model of micro-slotted dielectric elastomer bending actuators (MSDEBAs). One-quarter of the symmetrical unit (in blue) showing is just an extraction of the rectangular unimorph at: (**a**) inactive state; (**b**) activated states; (**c**) f–f’ cross-sectional side view of the bilayer unimorph. (note: *L* is the height of the triangular petal assuming fixed side as the base, *R* is the radius of curvature of the activated petal, *d* is the slit-width, E_1_, v_1_ and *t*_*1*_ are the Elastic modulus, Poisson’s ratio and thickness of dielectric elastomer; E_2_, v_2_ and *t*_*2*_ are the same for Polyethylene terephthalate (PET) films).

Figure 2 shows upon activation, the multi-layered MSDEBA bends like a composite unimorph with two distinct layer stiffnesses. It consists of the stiff mylar layer of thickness *t*_*1*_ = 25 µm and an equivalent soft layer with a thickness of 750 µm made of the 3 stacks of soft elastomer layers (VHB) each of thickness *t*_*2*_ = 250 µm. The VHB layers make the DEA. Each MSDEBA unit consists of four dielectric elastomer petals which are triangular-shaped dielectric elastomer bending actuators separated by the slit of width *d* = *0.36* *mm*. The two perpendicular edges of each triangular segment are free while the third edge is completely constrained. Hence, as shown in Fig. 2a, b, they behave like a rectangular unimorph since they are similar to a triangular section extracted from the rectangular section with one constrained edge. When it is activated with a voltage *V*, the intended unidirectional actuation strain $(\frac{\Delta L}{{L}_{o}})$ of the DEA is given by,

1 $$\frac{\Delta L}{{L}_{o}}=\frac{\nu \epsilon {\epsilon }_{o}}{{E}_{2}}{\left(\frac{V}{t}\right)}^{2}$$

where, *t* is the thickness of the individual layers of the VHB substrate, ν is the Poisson ratio, ${\epsilon }_{o}$ is the dielectric constant, and ${E}_{2}$ is Young’s modulus of the VHB substrate. Since the stiff mylar layer and the VHB layer are bonded, the induced in-plane stress causes the system to bend like the bimetallic unimorph as described by Timoshenko et al.^56^ (see Fig. 2c). The radius of curvature of such a bending system (see Fig. 2c) is given by Timoshenko et al.^56,57^ as,

2 $$\frac{1}{R}=\frac{\Delta L}{{L}_{o}}* \left(\frac{1}{\frac{{t}_{1}+{t}_{2}}{2}+\frac{2}{{t}_{1}+{t}_{2}}\left(\frac{1}{{E}_{1}{t}_{1}}+\frac{1}{{E}_{2}{t}_{2}}\right)\left(\frac{{E}_{1}{t}_{1}^{3}+{E}_{2}{t}_{2}^{3}}{12}\right)}\right)$$

The acoustic property of the MSDEBA absorbers depends on the open ratio and the micro-slot gaps (G). Supplementary Note 2 shows a detailed Electromechanical model of the MSDEBA based on geometrical model of the micro-slots of MSDEBA shown in Supplementary Fig. 2d–f. Figure 2a, b shows that at the inactive state, the MSDEBA segments lie flat with a constant slit gap (*d*). Meanwhile, at the activated states, the MSDEBA bends, and it obtains a shape like a triangle wrapped around a cylinder of radius R. Due to the unique design of MSDEBA, the slit-gap increases but is not constant from the corner to the centre of the MSDEBA. The gap between the adjacent free tips of the MSDEBA unit is derived to be,

3 $${G}_{x=0}=d+\sqrt{2}* \left\{L-\frac{d}{\sqrt{2}}-R* \sin \left(\frac{L-\frac{d}{\sqrt{2}}}{R}\right)\right\}$$

Here *L* is the half-length of the square formed by the ends of the cross-shaped micro-slots in each unit of MSDEBA. The projected open area of each unit of MSDEBA at the inactive state *A*_*v* *=* *0*_ is equal to $2\sqrt{2}L* d$. The total projected open area *A*_*v*_ at the activated state is given by,

4 $${A}_{v}=2\sqrt{2}L* d+8* \left\{\frac{{\left(L-d/{\sqrt{2}}\right)}^{2}}{2}-{R}^{2}+{R}^{2}* \cos \left(\frac{L-\frac{d}{\sqrt{2}}}{R}\right)\right\}$$

Then the voltage-dependent projected open ratio $(\phi (V))$ is given by *A*_*v*_*/b*^*2*^, where *b*^*2*^ is the area of the square MSDEBA units. This model was validated by FEM simulation and experiments.

### Electrical activation performance

**Fig. 3:**
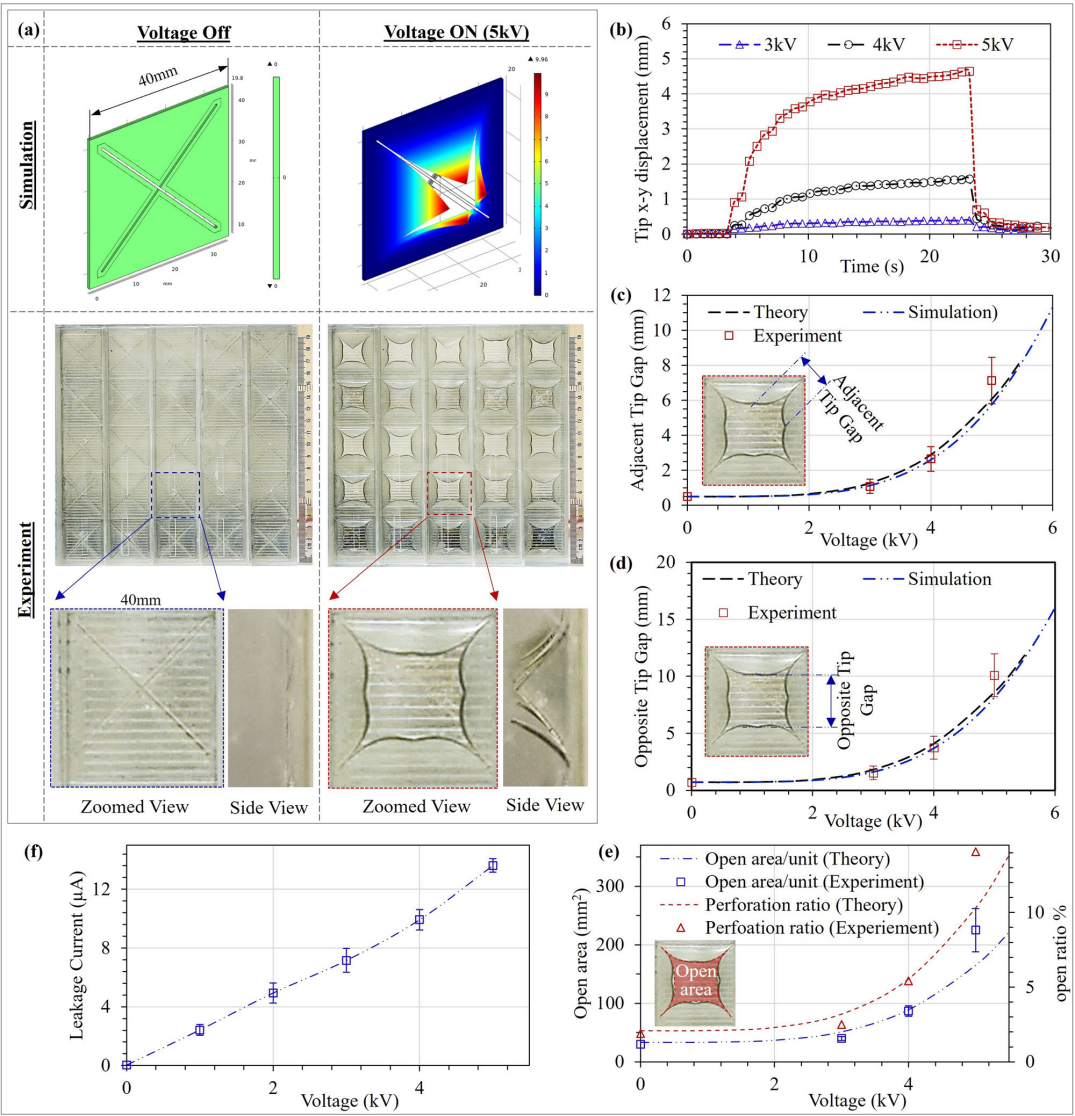
Analytical, Finite element analysis (FEA) simulation and experimental results showing bending actuation and opening of the micro-slotted dielectric elastomer bending actuators (MSDEBAs). **a** Pictures of cross-shaped slotted MSDEBA at the inactive and activated states. **b** Plots for the in-plane displacement of the quadrant’s free tips upon electrical activation. **c** Distance between the adjacent tips and **d** distance between opposite tips for the cross-slotted MSDEBA. **e** Projected open area and perforation ratio of the MSDEBA. **f** Electrical current leaking through the MSDEBA at activated states indicating power consumption. (Note: Error bars are calculated using standard deviation of measurements for three samples).

The reported opening motion of reconfigurable MSDEBA-based acoustic absorber upon high voltage activation is validated through analytical models, FEM simulation, and experiments. Figure 3 shows the petal motion-like bending actuation of the four dielectric elastomer petals of an MSDEBA unit which increases the open ratio of the absorber. At inactive state, all dielectric elastomer petals are flat, thus the MSDEBA layer covers most of the parallel micro-slots in the acrylic MSP. The gap between the adjacent tips and the constant slit-width of the MSDEBA units is 0.36 µm. Hence, the calculated open area per unit MSDEBA is 32.5 mm^2^ and the overall open ratio is 2%. As the voltage applied to the MSDEBA is increased, the dielectric elastomer petals start to bend (see Fig. 3a). This bending causes the dielectric elastomer petals’ free tips at the centre of the MSDEBA to move apart leading to an increase in the projected open area and thus increasing the open ratio of the absorber. Experimental results of multiple specimens show that at a voltage of 5 kV, the triangular segment of the dielectric elastomer petals bend making a radius of curvature of 12.14 mm. Consequently, the adjacent tips move apart by 7.13 ± 1.33 mm and the open ratio of the MSDEBA layer increases to 14.07%. Figure 3b–e shows that these experimental results closely match the presented analytical bending model and the FEA simulation (also refer to Supplementary Video 4 that shows simulated opening of a MSDEBA unit). As shown in Fig. 3f, the current leaking through the MSDEBA at 5 kV activation is 13.62 µA. Hence at the most power-consuming state, the absorber will consume 1.70 watts/m^2^. Deactivation allows the MSDEBA segments to reach their original flat state. This device has a response time of ~12 s to reach 90% of its final activated state and approximately a second to reach its flat deactivated state. The non-linear viscoelastic nature of acrylic elastomer used in the MSDEBA is the main cause for the viscoelastic drift that slows the response speed during activation^58,59^. In addition, such viscoelastic elastomer shows significant hysteresis (strain vs electric field), especially at a low frequency that causes variation in the relaxation time. This effect resulted in a variation in the response speed during the activation and deactivation of the MSDEBAs^58–60^.

### Acoustic model

#### Parallel-arranged varying depth back cavities

**Fig. 4:**
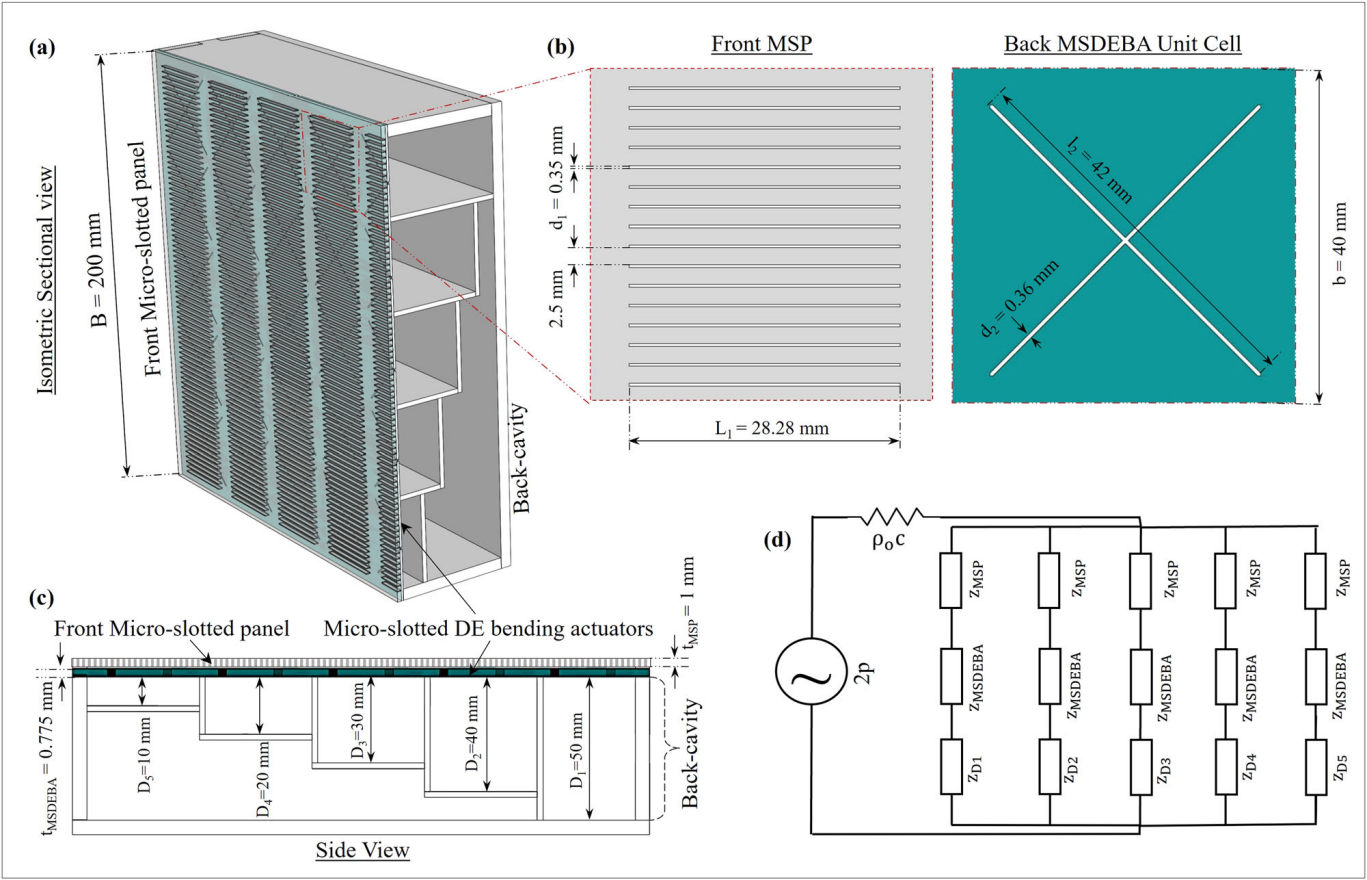
Acoustic Model of the micro-slotted dielectric elastomer bending actuator (MSDEBA)-based absorber. **a** Cross-sectional isometric view of a unit acoustic absorber with parallel-arranged varying depth back cavity. **b** Enlarged view of the Front micro-slotted panel (MSP) and the back MSDEBA. **c** Side view of the unit acoustic absorber box. **d** Electro-acoustic model of the MSP/MSDEBA/varying-depth (VD) back cavity-based acoustic absorber. (Note: B is the width and height of the absorber panel prototypes, d_1_ slit-width of the MSP, d_2_ is the slit-width of the MSDEBA, b is the length/width of each unit of the MSDEBA, D_1-5_ is the depth of each back-cavity).

To obtain broadband acoustic absorption, a straightforward approach is to arrange multiple MPPs of different frequency characteristics in parallel, forming an MPP absorber array. It seems natural that broader absorption bandwidth can be achieved by combining different frequency bands. Therefore, a simple approach is to parallelly arrange back cavities of varying depth^61^ as shown in Fig. 4a–c. Wang et al.^62^ studied the micro-perforated panel absorbers with parallel arranged varying depth back cavities. Their study concluded that strong local resonance occurs within the MPP absorber array due to the different reactance matching conditions of each MPP absorber component having different back cavity depths. As such, most of the acoustic energy is attracted toward and absorbed by the resonating MPP absorber component. The supplementary sound absorption by the non-resonating MPP absorber component is trivial. Also, the out-of-phase air motion changes the effective acoustic reactance of the MPP patch covering the resonating cavity, which shifts the resonance frequency. These three aspects, namely: the strong local resonance absorption; the supplementary absorption by the non-resonating absorbers; and the change of environmental impedance conditions, constitute the parallel absorption mechanism. This parallel absorption mechanism also applies to micro-slotted panels (MSP). Supplementary Note 1 shows a comparison of the simulated acoustic performance of MSP absorbers with a constant depth back cavity and the parallel arranged varying depth back cavity. Supplementary Fig. 1 shows a parallelly arranged varying depth back cavity can extend the absorption bandwidth. Due to constraints from acoustic measurement setup and commercial application requirements, the overall dimension of the unit absorber was set to be 200 mm x 200 mm x 50 mm. Numerical and experiment tests were done for over 40 cases with variations of D1 to D5 (i.e., depths of the parallel back-cavities) while keeping their widths equal and constant. Eventually, the parallel-arranged back-cavity depths D1 to D5 that gave optimal and broad absorption at lower frequencies were selected. In the current work, the parallel back cavity consists of five different depths, namely: D_1_ = 50 mm, D_2_ = 40 mm, D_3_ = 30 mm, D_4_ = 20 mm, and D_5_ = 10 mm.

#### Acoustic model of MSDEBA-based sound absorber

Figure 4a shows the MSDEBA-based sound absorber consisting of front MSP, back MSDEBA (micro-slotted bending actuator) and parallel back cavities of various depths. Assuming all the back-cavity compartments are of equal width (*b*) and are covered by micro-slotted panels of the same dimensions, Fig. 4d shows the equivalent electro-acoustic model of the MSDEBA-based sound absorber. The admittance or 1/(total impedance) of such absorber is given by^62^:

5 $$\frac{1}{Z}=\mathop{\sum }\limits_{x=1}^{5}\frac{b/B}{{Z}_{{MSP}}+Z({D}_{x}){+Z}_{{MSDEBA}}}$$

Here, ${D}_{x}(x=1\ldots 5)$ are the depths of the sub-chambers, the acoustic impedance of the micro-slotted front panel is$\,{Z}_{{MSP}}$, and that of micro-slotted dielectric elastomer bending actuator is ${Z}_{{MSDEBA}}$. ${Z}_{{MSP}}$ is given by the Equivalent Fluid (EF) model as^63,64^:

6 $${Z}_{{MSP}}=\frac{i\omega {\rho }_{o}{t}_{1}}{\phi }\left\{{\left[1-\frac{\tanh (s\sqrt{i})}{(s\sqrt{i})}\right]}^{-1}-\frac{2{d}_{1}}{\pi {t}_{1}}{{{{\mathrm{ln}}}}}\left[\sin \left(\frac{\pi \phi }{2}\right)\right]-i\frac{{d}_{\nu }}{{t}_{1}}\right\}$$

where, $s=d\sqrt{\frac{\omega {\rho }_{o}}{4\eta }}$, ${d}_{\nu }=\sqrt{\frac{2\eta }{\omega {\rho }_{o}}}$ and perforation ratio $\phi =\frac{{total}\,{slit}\,{area}}{{Total}\,{area}}=\frac{n* {d}_{1}* {l}_{1}}{{B}^{2}}$. Here, *n* is the number of micro slots in the MSP, *d*_*1*_ is the slit width, *l*_*1*_ is the length of the micro-slots, *t*_*1*_ is MSP thickness, ${\rho }_{o}$ is the air density, $\eta$ is air viscosity and $\omega$ is the angular frequency. Similarly, ${Z}_{{MSDEBA}}$ is given by:

7 $${Z}_{{MSDEBA}}=\frac{i\omega {\rho }_{o}{t}_{2}}{\phi (V)}\left\{{\left[1-\frac{\tanh (s\sqrt{i})}{(s\sqrt{i})}\right]}^{-1} -\frac{2{d}_{2}}{\pi {t}_{2}}{{{{\mathrm{ln}}}}}\left[\sin \left(\frac{\pi \phi (V)}{2}\right)\right]-i\frac{{d}_{\nu }}{{t}_{2}}\right\}$$

where, the voltage-dependent perforation ratio of the MSDEBA is $\phi (V)=\frac{{total}\,{slit}\,{area}}{{Total}\,{area}}$, *d*_*2*_ is the slit width, and *t*_*2*_ is MSDEBA thickness. The inward impedance of the surface of the back cavities with depth (*D*_*x*_) is^65^:

8 $$Z({D}_{x})=\frac{{ic}{\rho }_{e}{k}_{o}}{{k}_{t}}\left(\cot \left({k}_{t}{D}_{x}\right)\right)$$

where, ${\rho }_{e}=\rho \left(\right.1+(1-i)\,\frac{{d}_{\nu }}{b}$, ${k}_{t}={k}_{o}+\frac{{k}_{o}}{2b}(1-i)(\,{d}_{\nu }+\left(\gamma -1\right){d}_{h})$, ${k}_{o}=\omega /c$, $\gamma$ is the ratio of the specific heat and is 1.4 for air, ${d}_{h}=\sqrt{\frac{2K}{\omega {\rho }_{o}{c}_{p}}}\approx 0.25\frac{1}{\sqrt{f}}$ is the thickness of the thermal boundary, K is thermal conductivity and ${c}_{p}$ is the heat capacity of the air. This model indicates that reconfiguration of the open ratio of the MSDEBA can be used to change the absorption characteristics of the presented absorber. Based on the electroacoustic model in Fig. 4d, the addition of MSP to the MSDEBA layer increases the acoustic resistance as well as the reactance of the absorber. This is manifested by the occurrence of the resonant peaks at lower frequencies in the absorption spectrum and a slight reduction of the absorption coefficient at those resonant frequencies (refer to supplementary file’s Supplementary Fig. 3f). Meanwhile for the combined reconfigurable system, based on Eqs. (1, 2 and 4) the projected open ratio (*ϕ*(*V*)) = A_V_/b^2^ has a quartic increase with the increase in the external control voltage as shown in Fig. 3e, which increases the overall porosity of the MSP/MSDEBA panel. As the open ratio (*V*) is increased, like any MPP or combination of MPPs^66–68^, it reduces the acoustic reactance of the system following Eqs. 5 and 7. Consequently, the resonant absorption peaks are shifted to higher frequencies. This suggests, that with a higher voltage causing a larger opening of the MSDEBA, a larger frequency shift will be achieved.

### Tunability of acoustic absorption spectrum

To demonstrate the tunability of the acoustic absorption spectrum of the MSDEBA-based acoustic absorber made of front MSP, MSDEBA, and parallel-arranged VD back-cavity were assembled. The combination of an MSP with an MSDEBA as front covers allows better acoustic absorption at the lower frequency range of 400-850 Hz compared to only MSP or only MSEDBA front covers. On the other hand, VD back-cavity enhances the bandwidth of the acoustic absorption spectrum. The contribution of MSP, MSDEBA and parallel-arranged VD back-cavity to acoustic absorption performance is further discussed in the Supplementary Note 3 in the supplementary files. The absorption performance of MSDEBA-based absorbers and their tunable feature are discussed henceforth.

**Fig. 5:**
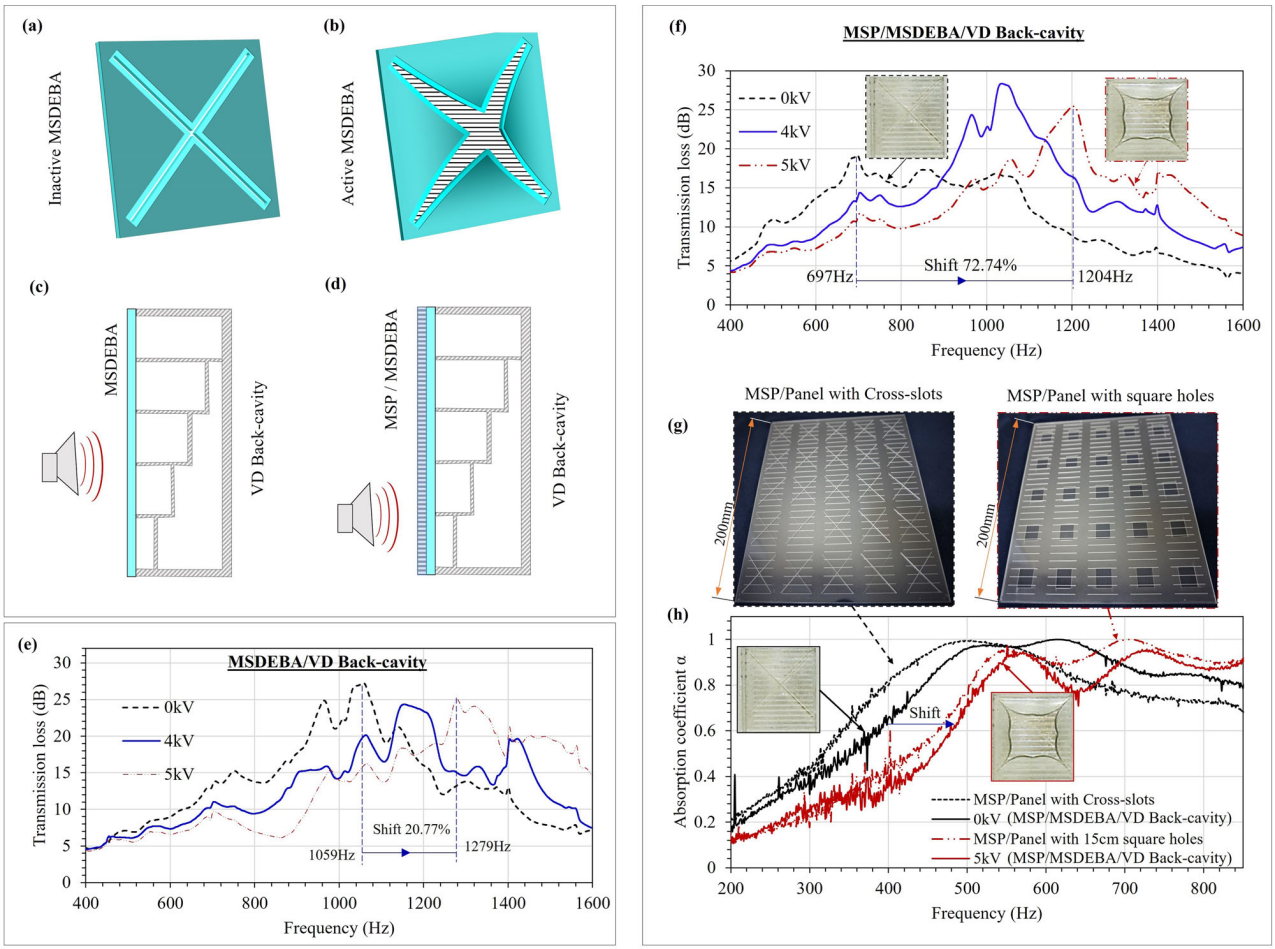
Tuning of acoustic absorption spectrum by reconfiguring the absorber through micro-slotted dielectric elastomer bending actuator (MSDEBA) activation. **a** Schematic of inactive and (**b**) active state of MSDEBA; Schematics of the acoustic absorber with (**c**) MSDEBA/ Varying-depth (VD) back-cavity; (**d**) Front micro-slotted panel (MSP)/Back MSDEBA/VD back-cavity. Comparison of acoustic transmission loss (**e**) for absorber with MSDEBA/VD Back-cavity at different activation voltage; (**f**) for absorber with Front MSP/ Back MSDEBA/VD Back-cavity at different activation voltage; (**g**) Photos of the 1 mm thick acrylic panel with cross-slots and square holes placed behind the MSP to mimic MSDEBA at 0 kV and 5 kV respectively. **h** Normal incidence absorption coefficient of absorber with Front MSP/back MSDEBA/VD Back-cavity at 0 kV and 5 kV and comparison with the mimicking panels with cross-slots and square holes.

An MSDEBA can be activated to open up (see Fig. 5a, b and tune the peak absorption spectrum to higher frequencies). Hence, this tunability allows the MSDEBA absorbers to actively target the noise in a wider frequency range compared to their passive counterparts. In addition, as lower-frequency noise can travel further and is more difficult to attenuate, an acoustic absorber set to absorb lower-frequency noise at the default inactive state could be more desirable. The combination of MSP, MSDEBA and VD back-cavity (see Fig. 5c, d) forms an absorber that has > 15 dB transmission from ~640 Hz to 1075 Hz and a peak absorption of 19 dB at 697 Hz. High voltage activation of the MSDEBA increases the open ratio of the MSDEBA layer. Consequently, the peak absorption will occur at a higher frequency. Figure 5e shows the 5 kV activation of the MSDEBA/VD back-cavity absorber shifted the frequency at which peak transmission loss occurs by 20.77% (i.e., from 1059 Hz to 1279 Hz). Such tunability doubled the 15 dB absorption bandwidth of these absorbers. At inactive state, they had 15 dB bandwidth of ~365 Hz, which is increased to 740 Hz (850 Hz to 1590 Hz) due to acoustic spectrum tuning capability. As the activation of the MSDEBA shifts the acoustic spectrum to the higher frequency, designing the inactive state acoustic spectrum to be at a lower frequency by the addition of the front MSP to the MSDEBA/VD back-cavity absorber makes a more tunable and broadband absorber.

Figure 5f shows the 5 kV activation of the MSP/MSDEBA/VD back-cavity absorber shifted the frequency at which peak transmission loss occurs by 72.74% (i.e., from 697 Hz to 1204 Hz). This tunability doubled the 15 dB absorption bandwidth of these absorbers. At inactive state, they had 15 dB bandwidth of ~435 Hz, which is increased to 820 Hz (640–1460 Hz) due to acoustic spectrum tuning capability. To simply explain the tunable characteristic of the MSP/MSDEBA/VD back-cavity absorber, 1 mm thick acrylic panels with cross-slots and square holes mimicking the open ratio of MSDEBA at inactive and activated state respectively were made and assembled with MSP and VD back-cavity as shown in Fig. 5g.

**Fig. 6:**
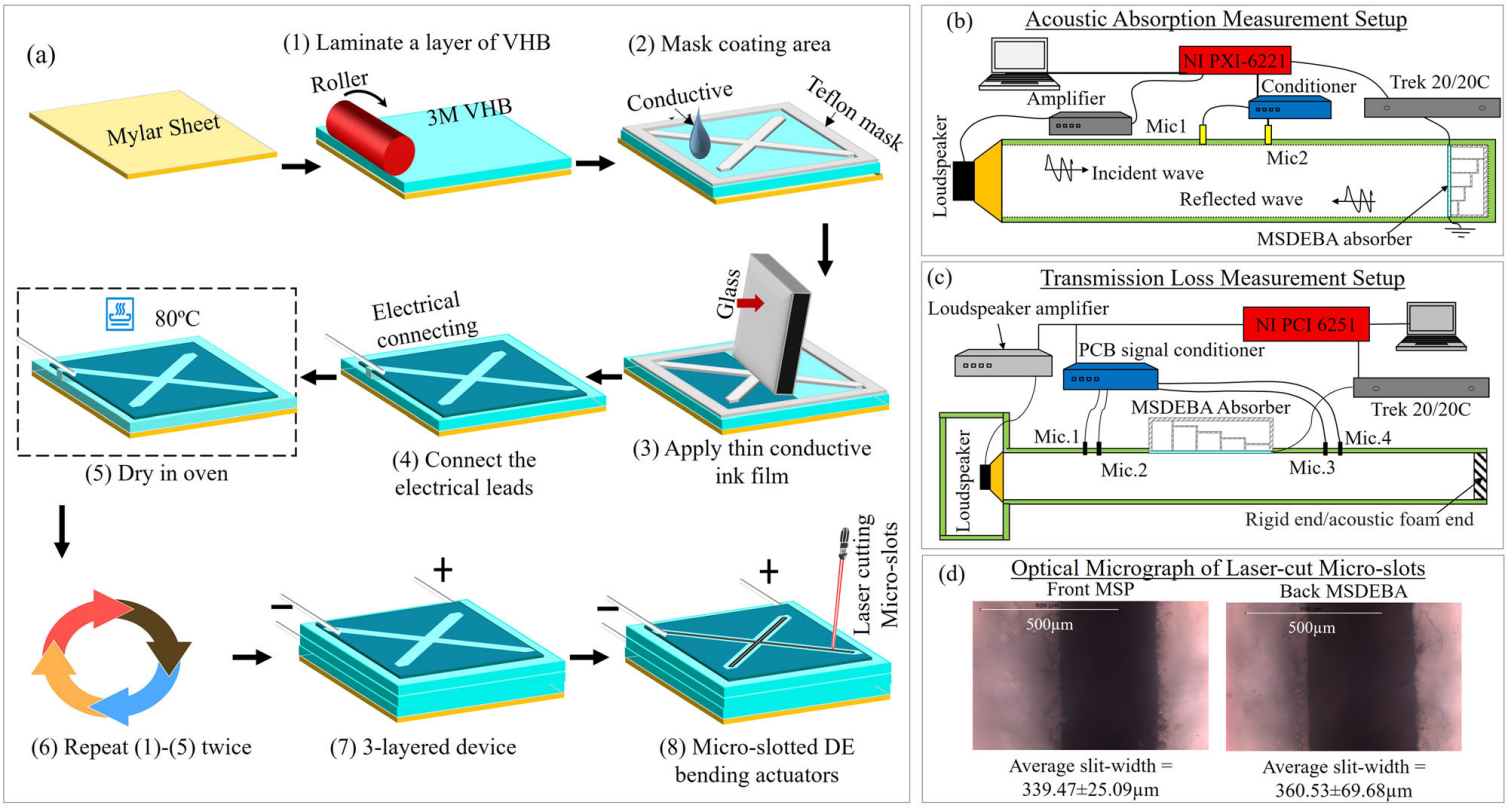
Fabrication procedure and acoustic measurement setup for the tunable acoustic absorbers. **a** Fabrication procedure of the multi-layered micro-slotted dielectric elastomer bending actuators (MSDEBAs). **b** Measurement setup for the normal incident absorption coefficient. **c** Measurement setup for the acoustic transmission loss. **d** Optical micrograph of the micro-slots formed by laser cutting.

Figure 5h compares the normal incidence absorption coefficient of acoustic absorber with MSP/MSDEBA/VD Back-cavity absorbers at inactive and 5 kV activated states with absorbers replacing MSDEBA by mimicking panels having cross-slots and square holes respectively. The absorption spectrums of the MSP/MSDEBA/VD back-cavity absorber at the inactive state (i.e., 0 kV) are close to the spectrum of the absorber that replaced MSDEBA with cross-slot mimicking panels. Meanwhile, the absorption spectrums of the MSP/MSDEBA/VD back-cavity absorber at a 5 kV activated state are close to the spectrum of the absorber that replaced MSDEBA with mimicking panels having square holes. This comparison of spectrums in Fig. 5h allows us to conclude that the acoustic spectrum tuning capabilities of the MSDEBA-based absorber are mostly attributed to its capability of actively controlling the open ratio of the MSDEBA. The absorption coefficient that was measured with a normal incidence of sound and the transmission loss that was measured with a parallel incidence (180°) (see Fig. 6) suggests the proposed absorber could also be used to attenuate sound and tune the absorption peaks at various angle of incidence of the sound.

## Methods

### Actuator’s fabrication

The MSDEBA is fabricated through a layer-by-layer process. It consists of a layer of 25 µm thick PET film (Mylar) bonded to 3 layers of 250 µm thick VHB elastomer membranes (3 M VHB F9469PC). Each VHB layer is sandwiched between the patterned transparent electrode film made of AgNW/PEDOT:PSS. Figure 6a shows the schematic of the process involved in the fabrication of the MSDEBA device. First, a layer of PET film is coated with a VHB layer by carefully rolling the VHB with liners onto the PET film. Secondly, after peeling off the liners, a laser-cut mask made of Teflon films is arranged on the VHB. The ink is then coated onto the masked VHB using the doctor-blading method. Tin-coated copper wire of 50 µm is placed parallelly over the ink-coated part to act as electrical leads and a uniform charge distributor. Then the ink is cured at 80 °C for about 1 h. Similarly, the process is repeated to layer another VHB membrane and conductive ink and wire in the same sequence until there are 3 layers of VHB and patterned electrodes. Special care is taken to align each of the patterned electrodes. Finally, cross-shaped micro-slots are drilled in the MSDEBA using a laser cutting process (Epilog laser cutting machine 60 W). The laser-cut micro-slots of 360 µm slit-width are aligned to form at the centre of the cross-shaped non-coated portion of the patterned electrodes. Later the MSP and MSDEBA layers are placed together with the PET layer facing outward. They are then assembled with the varying depth back cavity which is also fabricated by laser-cutting and assembling of the 2 mm and 5 mm thick acrylic panels.

### Characterization of MSDEBA actuation

The bending actuation of the MSDEBA is characterized using a setup consisting of a high voltage power supply (Trek 20/20 C), NI data logger (NI PCI 6251) and a digital camera (Canon D7000). LabVIEW program is used to control the voltage applied to the MSDEBA and the activation duration. Videos of the MSDEBA are taken during the activation and later Tracker software is used to track the motion of the tips of the MSDEBA which is marked by a black dot (Refer to Supplementary Videos 1–3). Using the tracked data point the adjacent tip distance and opposite tip distance are calculated, which are the indication of the opening of the MSDEBA.

### FEA Simulation of MSDEBA

COMSOL Multiphysics® Version 5.5 software was used for the electromechanical 3D simulation of the MSDEBA bending actuation. The structural module and the electromechanical module were coupled together to simulate the bending motion of the MSDEBA upon electrical activation. CAD model of the MSDEBA with its 1 layer of 25 µm PET layer and 3 layers of 250 µm thick VHB layer were assembled in Solidworks software. The CAD model is a replica of the actual device with the passive edges near the micro-slots as shown in Fig. 1c and Supplementary Fig. 1d. The four edges of the MSDEBA unit were completely constrained and the outermost and interlayer of the VHB were allocated with ground and bias voltages to replicate a DEA. As the deformation involved is large, a non-linear geometrical simulation in conjunction with parametric sweep replicating gradually increasing voltage was done. As for the structural parameters, the PET layer is taken as an elastic material with an elastic modulus of 4.13 GPa. The VHB was represented by the Mooney-Rivlin hyperelastic material model with Young’s modulus of 220kPa, Poisson’s ratio of 0.49, and Mooney-Rivlin model parameters: C_10_ = 9888.3 Pa, C_01_ = 33089.1 Pa^69^. The relative permittivity and density of VHB were taken as 4.7 and 960 kg/m^3^ respectively.

### Acoustic measurement

The acoustic absorption performance of the MSDEBA absorber is measured at various activation voltages. To study the performance of the actuator at various sound incidence conditions two acoustic measurement setups are used.

### Absorption coefficient measurement

Figure 6b shows an acoustic impedance tube being used to measure the acoustic absorption spectrum of a tunable absorber at normal incidence. The 500 mm long duct with a cross-section of$\,200{{{{{\rm{mm}}}}}}\times 200{{{{{\rm{mm}}}}}}$ has a loudspeaker installed at one end and the tunable absorber mounted at the other end. Two electret array microphones (PCB piezotronic, model 130E20) were used to measure the sound pressures in the tube, namely, *p*_*i*_ and *p*_*r*_ of the incident and reflected sound waves, respectively. According to ref. ^70^, the sound absorption coefficient (α) is calculated as$1-{\left|\frac{pr}{pi}\right|}^{2}$. At a 20 mm distance between them, the two microphones can measure the sound pressure down to 200 Hz. During the acoustic testing, a data logger NI PXI 6221 was used for data recording, whereas a high-voltage amplifier (Trek model 20/20 C) was used for driving a device of MSDEBA.

### Transmission loss measurement

The acoustic measurement was conducted in the rectangle duct with a cross-section of$\,200{{{{{\rm{mm}}}}}}\times 40{{{{{\rm{mm}}}}}}$; the whole system is shown in Fig. 6c. A loudspeaker is installed at one end of the duct and acts as the sound source for generating sine waves or the white noise used in the measurement. Four PCB array microphones model 130E20 were used for measuring the sound pressure inside the duct. These microphones are referred to as ‘Mic.1’, ‘Mic.2’, ‘Mic.3’ and ‘Mic.4’ in Fig. 6c. The two-load method is used to measure the transmission loss (TL) of the MSDEBA-base silencer^71^, one of the advantages of this method is that it does not need a complete absorption anechoic end, only the normal rigid and acoustic foam end can be used for the transmission loss measurements. The frequency range of the present duct is from 50 Hz to 1700Hz due to the dimensions of the cross-section of the duct. All the acquisition and control signals were conducted based on the NI PCI platform. It also needs to be noted that the sampling frequency was set to 40 kHz and the record time is 4 s to ensure a smaller $\Delta f$ in the FFT analysis. In the present experiment, Mic.1 was chosen as the referred microphone of the system, the magnitudes and phases of the other three microphones were then calibrated with Mic.1^71^. Additionally, the distances between Mic.1 and Mic.2 and between Mic.3 and Mic.4 were set to 30 mm, so the lower limit of the measurement frequency of the system is about 100 Hz.

### Supplementary information

Peer Review File

Supplemental Information

Description of Additional Supplementary Files

Supplementary video 1

Supplementary video 2

Supplementary video 3

Supplementary video 4

## Data Availability

All relevant data are available from the authors upon request and will be provided by M. Shrestha (E-mail: milan001@e.ntu.edu.sg).
