# Supplementary material for: A tunable acoustic absorber using reconfigurable dielectric elastomer actuated petals
Source: Commun Eng. 2024 Jan 10;3:11. doi: 10.1038/s44172-023-00159-z (PMC10955946; doi:10.1038/s44172-023-00159-z)
Supplement: Supplementary file 2 — Supplemental Information [file 44172_2023_159_MOESM2_ESM.pdf]

Title: A tunable acoustic absorber using reconfigurable dielectric elastomer actuated petals
Authors: M. Shrestha[1,2*], G.K. Lau[3], Y.W. Chin[2], E.H.T. Teo[4,5], B.C. Khoo[2], Z. LU[6*]

Affiliations

[1*] Continental-NTU Corporate Lab, Nanyang Technological University, Singapore 639798, Singapore. milan001@e.ntu.edu.sg

[2] National University of Singapore, Singapore 117411, Singapore.

[3] Department of Mechanical Engineering, National Yang Ming Chiao Tung University, Hsinchu 300093, Taiwan.

[4] School of Electrical and Electronic Engineering, Nanyang Technological University, Singapore 639798, Singapore.

[5] School of Materials Science and Engineering, Nanyang Technological University, Singapore 639798, Singapore.

[6*] School of Aeronautics and Astronoautics, Sun Yat-Sen University, Shenzhen 518107, PR China. luzhb7@mail.sysu.edu.cn

Supplementary Information

Supplementary Note 1. Finite element analysis (FEA) simulation of Parallel-arranged varying depth back cavities

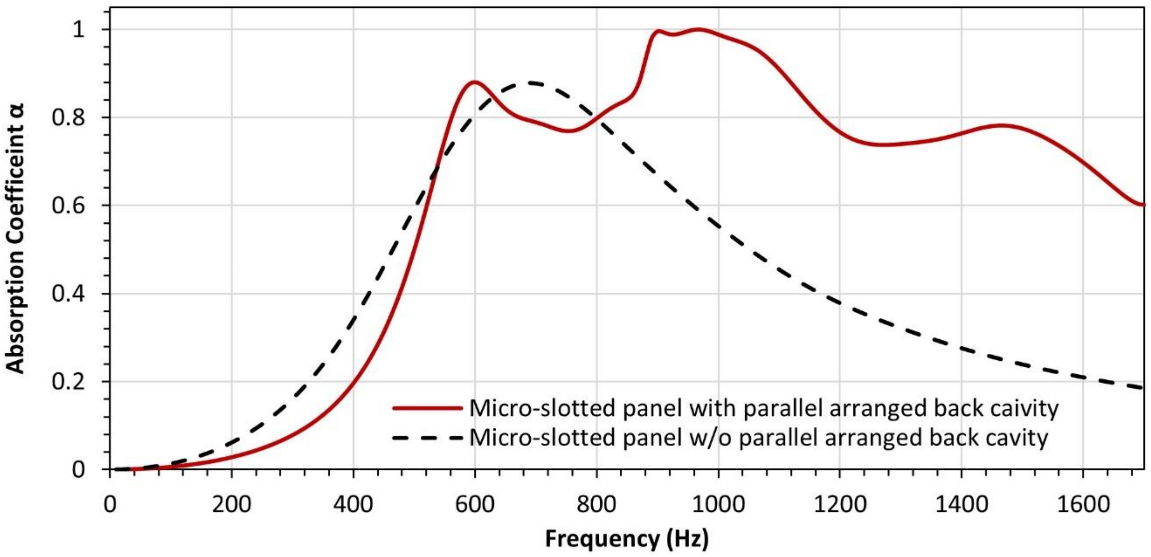

Supplementary Figure 1. Acoustic performance comparison of Micro-slotted absorbers with parallel arranged varying depth back cavity and with a constant depth back cavity.

Supplementary Note 2. Derivation of Electromechanical model of the Micro-slotted Dielectric Elastomer Bending Actuator

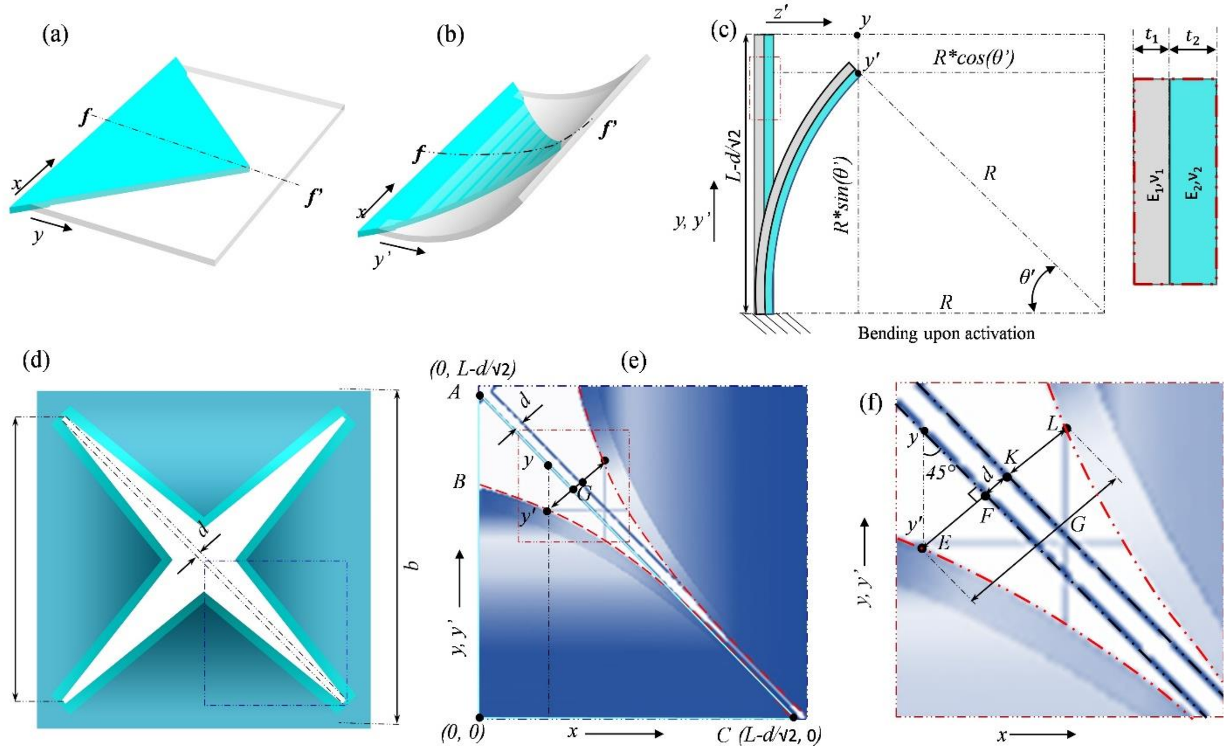

Supplementary Figure 2. Electromechanical model of micro-slotted dielectric elastomer bending actuators (MSDEBAs). One-quarter of the symmetrical unit (in blue) showing is just an extraction of the rectangular unimorph(a) inactive state. (b) activated states; (c) f-f' cross-sectional side view of the bilayer unimorph; (d) front view of one unit of the MSDEBA with active and inactive states overlapped (e) zoomed view of one quadrant of a unit cell; (f) geometric representation of the cross-section of the bent component.

The MSDEBA absorber is made of multiple layers. From a structural perspective, it consists of a stiff mylar layer of thickness t_1 and a soft VHB layer of thickness t_2. The VHB layers make the DEA. The reported design of the MSDE bending actuator consists of four triangular-shaped DE bending actuators separated by the slit. Yet, they behave like a rectangular unimorph as they are similar to a triangular section extracted from the rectangular section as shown in Figure S2(a-b). When it is activated with a voltage V, the unidirectional actuation strain-induced is given by,

$$\frac{\Delta L}{L_o} = \frac{\nu \epsilon \epsilon_o}{E_2}\left(\frac{V}{t}\right)^2 \quad (1)$$

where t is the thickness of the individual layers of the VHB substrate. Since the stiff mylar layer and the VHB layer are bonded, this induced strain causes the system to bend like the bimetallic unimorph described by Timoshenko et. al.[40] (see Figure S2 (c)). The radius of curvature of such a bending system is given by Timoshenko et. al.[40] as,

$$\frac{1}{R} = \frac{\Delta L}{L_o} * \left(\frac{1}{\frac{t_1 + t_2}{2} + 2\left(\frac{1}{E_1 A_1} + \frac{1}{E_2 A_2}\right)\left(\frac{E_1 I_1 + E_2 I_2}{t_1 + t_2}\right)} \right) \quad (2)$$

$$\frac{1}{R} = \frac{\Delta L}{L_o} * \left(\frac{1}{\frac{t_1 + t_2}{2} + \frac{2}{t_1 + t_2}\left(\frac{1}{E_1 t_1} + \frac{1}{E_2 t_2}\right)\left(\frac{E_1 t_1^3 + E_2 t_2^3}{12}\right)} \right) \quad (3)$$

The acoustic property of the MSDEBA absorbers depends on the open ratio and the micro-slots gaps (G). Figure S2(d)) shows that the slit gap *(d)* is constant at the inactive state. But, due to the unique design of this MSDEBA, upon activation, the gap is not constant. Figure S2(e, f) shows the geometrical model of the micro-slots. In Figure S2(e)) the line AC represents the edge of the micro-slots before activation. Upon, electrical activation the actuators bend in the z-direction as shown in Figure S2(c)).

The edge in the front x-y plane is represented by the curve BC. The increment in the slit width and open area can be evaluated using these two lines. The equation of the straight-line AC passing through the points *(L-d/√2, 0) and (0, L-d/√2)* can be found in the (x, y) coordinate system as:

$$y = -x + L - d/\sqrt{2} \tag{4}$$

At the activated states, the MSDEBA bends and it obtains a shape similar to a triangle wrapped around a cylinder of radius R. The x coordinates of the edges can be assumed to remain constant. Figure S2(c-e) shows only the y and z coordinate changes and hence, y' and z' is used as the coordinates of the edges at the activated state. The centre angle made by the slit edges (θ') is the function of y coordinates of the edges at this active state. This angle (θ') is given by

$$\theta' = y/R \tag{5}$$

$$\theta' = \left(-x + L - d/\sqrt{2}\right)/R \tag{6}$$

Meanwhile, from Figure S2(d) the y and z-coordinates of the edge at the activated state are given by,

$$y' = R * sin(\theta') \ and \ z' = R * (1 - \cos(\theta')) \tag{7}$$

The x and y coordinate completely define the micro-slot edge geometry at the inactive state. The x, y' and z'-coordinates define the micro-slots edge geometry at the active state. The micro-slots edge displacement is given by,

$$\Delta_{tot} = \sqrt{(y - y')^2 + (z')^2} \tag{8}$$

Figure S2(c)) shows that the micro-slots gap at the active state is the summation of the inactive slit gap *(d)* and the line EF and KL. Also, $EF = KF = (y - y')/\sqrt{2}$. Therefore, the slit gap *(G)* can be derived as the function of the x-coordinate as follows,

$$G = d + 2 * (y - y')/\sqrt{2} \tag{9}$$

$$G = d + \sqrt{2} * \left\{-x + L - \frac{d}{\sqrt{2}} - R * \sin\left(\frac{-x + L - \frac{d}{\sqrt{2}}}{R}\right)\right\} \tag{10}$$

Therefore, the gap of the adjacent tips is given by,

$$G_{x=0} = d + \sqrt{2} * \left\{L - \frac{d}{\sqrt{2}} - R * \sin\left(\frac{L - \frac{d}{\sqrt{2}}}{R}\right)\right\} \tag{11}$$

The open area at the inactive state $A_{v=0}$ is equal to $2\sqrt{2}L * d$. The increment in the open area upon activation is 8 times the area between the curve AC, BC and the y-axis. Therefore, the total open area A_v is given by,

$$A_v = 2\sqrt{2}L * d + 8 * \int_{x=0}^{x=L-d/\sqrt{2}} (y - y')dx \tag{12}$$

$$A_v = 2\sqrt{2}L * d + 8 * \left\{\frac{\left(L - d/\sqrt{2}\right)^2}{2} - R^2 + R^2 * \cos\left(\frac{L - \frac{d}{\sqrt{2}}}{R}\right)\right\} \tag{13}$$

Then the open ratio is given by A_v/b^2.

Supplementary Note 3. Improvement of acoustic absorption spectrum through use of MSDEBA and the MSP in metasurface based acoustic absorber.

To demonstrate the contribution of various individual component of this acoustic metasurface to the absorption performance, four different combination of MSP, MSDEBA, Normal back cavity and VD back cavity were tested. First, the absorber with front MSP and back MSDEBA with a 50mm constant depth back-cavity (See Figure S3.B); Second, the absorber with front MSDEBA with a varying depth (VD) back-cavity (See Figure S3.C); Third, the absorber with front micro-slotted panel (MSP) with a varying depth (VD) back-cavity (See Figure S3.D). Finally, the absorber with front MSP, and back MSDEBA with the VD back-cavity (See Figure S3.E).

Figure S3.F shows the effect on acoustic absorption performance in terms of transmission loss and bandwidth by different configurations, for the frequency range of 400-1600 Hz. Firstly, the combination of a front MSP with a back MSDEBA front cover allows better acoustic absorption at the lower frequency range of 400-850Hz than only MSP or only MSEDBA front covers. Meanwhile, VD back-cavity enhances the acoustic absorption and the bandwidth compared to constant depth back-

cavity, with the same front cover combination of MSP/MSDEBA. The acoustic metasurface-based absorber made of MSP/MSDEBA and constant-depth back-cavity has approximately 10-13dB transmission loss in the measured frequency range, but with varying depth cavities, transmission loss is increased to 10-18dBa. Whereas absorbers having VD back-cavity with MSDEBA alone or MSP alone have much higher absorption peaks but at higher frequency ranges. For example, the absorber MSDEBA/VD back-cavity has 27dB absorption peaks but at 1059Hz and MSP/VD back-cavity has a 40dB absorption peak at 1508Hz. Therefore, the chosen configuration of MSP/MSDEBA/VD back cavity allows optimal absorption at low frequency in the default inactive states. The ability to reconfigure the open ratio of the MSDEBA hence can help the absorber to adapt to higher frequency noise if the need arises.

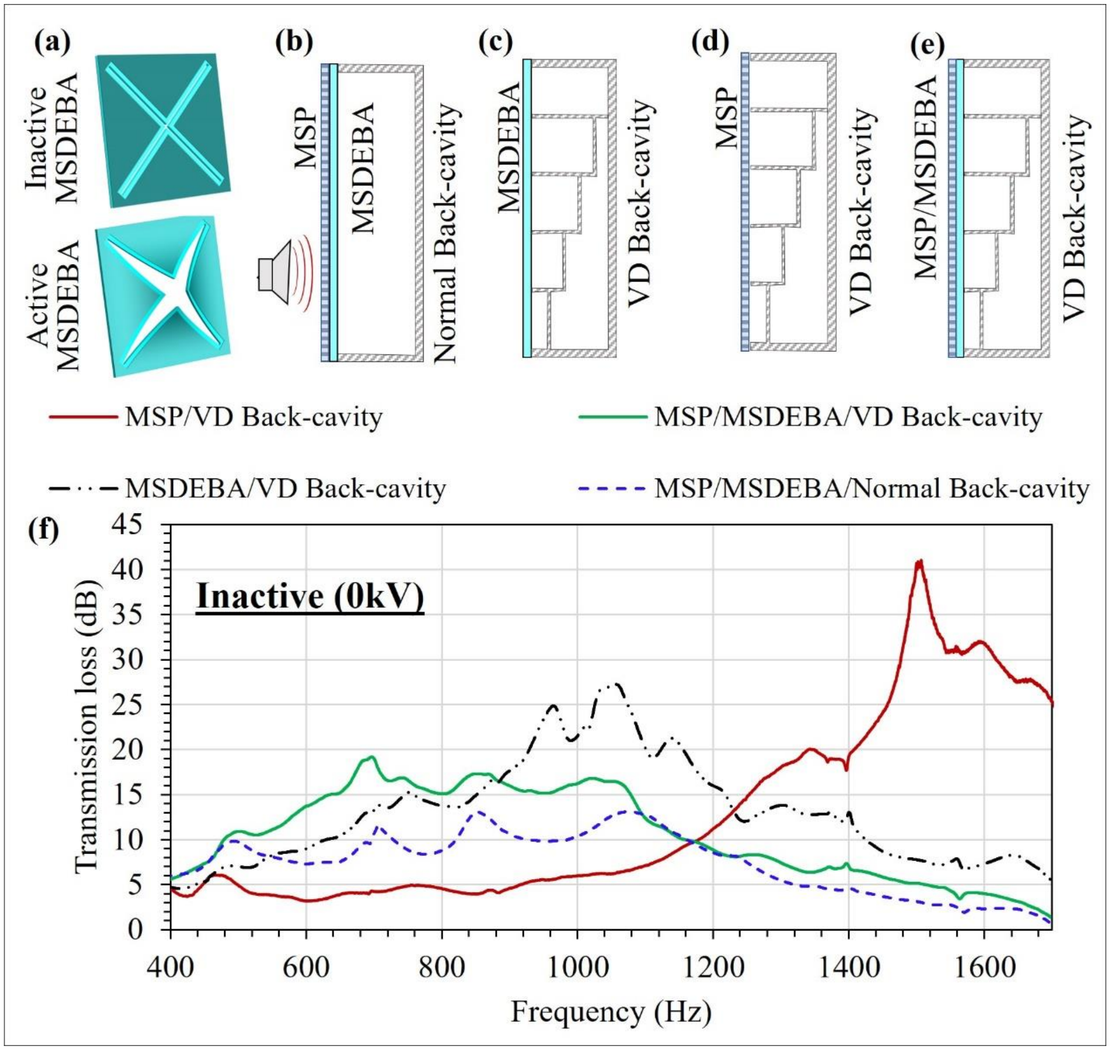

Supplementary Figure 3. Comparison of the effect of each component of the metasurface on absorption properties: (a) Schematic of active and inactive state of micro-slotted dielectric elastomer bending actuator (MSDEBA); Schematics of four type of absorbers focusing on effect of each component of the acoustic metasurface: (b) Front micro-slotted panel (MSP)/MSDEBA/Normal back-cavity; (c) MSDEBA/varying-depth (VD) back-cavity; (d) MSP/VD back-cavity; (e) Front MSP/Back MSDEBA/ VD back-cavity; (f) Comparison of acoustic transmission loss for the 4 types of acoustic absorbers at the inactive states (i.e., at 0kV).

<u>List of Supplementary Videos</u>

1. **Supplementary video 1: Activation of whole MSDEBA at 5KV (front View)**
2. **Supplementary video 2: Activation of whole MSDEBA at 5KV (Side View)**
3. **Supplementary video 3: Activation of single DE petal at 5kV (Isometric view)**
4. **Supplementary video 4: FEM simulated video of the unit of MSDEBA activated at 5kV**
