# Supplementary material for: A tunable acoustic absorber using reconfigurable dielectric elastomer actuated petals
Source: Commun Eng. 2024 Jan 10;3:11. doi: 10.1038/s44172-023-00159-z (PMC10955946; doi:10.1038/s44172-023-00159-z)
Supplement: Supplementary file 3 — Description of Additional Supplementary Files [file 44172_2023_159_MOESM3_ESM.pdf]

Description of Additional Supplementary Files

File name: Supplementary Video 1

Description: Activation of whole MSDEBA at 5KV (front View)

File name: Supplementary Video 2

Description: Activation of whole MSDEBA at 5KV (Side View)

File name: Supplementary Video 3

Description: Activation of single DE petal at 5kV (Isometric view)

File name: Supplementary Video 4

Description: FEM simulated video of the unit of MSDEBA activated at 5kV
