# Supplementary figures and images for: A tunable acoustic absorber using reconfigurable dielectric elastomer actuated petals
Source: Commun Eng. 2024 Jan 10;3:11. doi: 10.1038/s44172-023-00159-z (PMC10955946; doi:10.1038/s44172-023-00159-z)

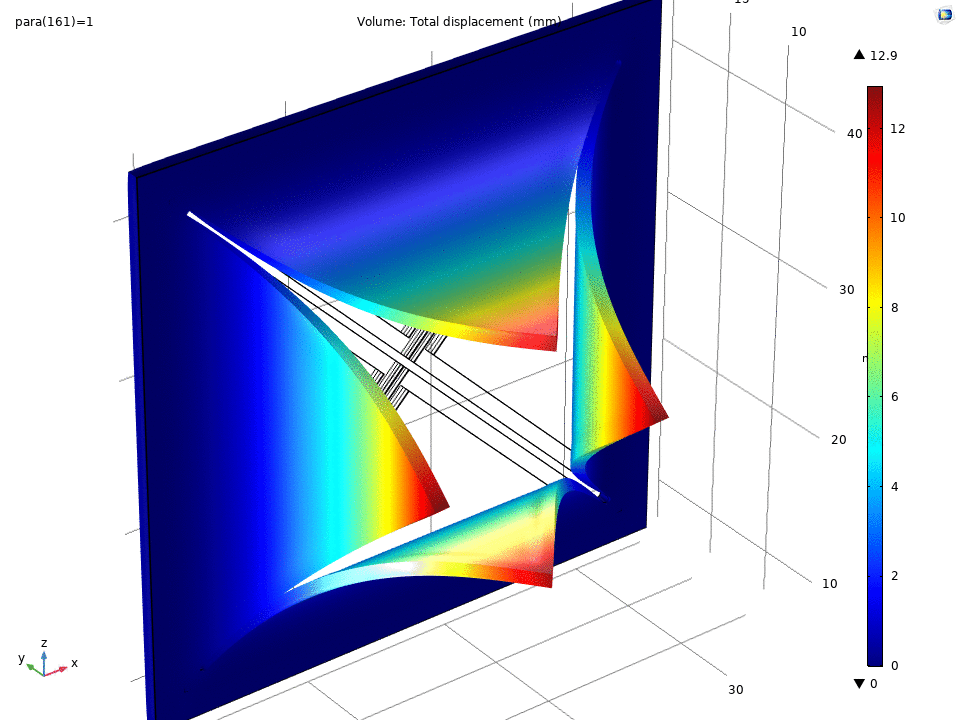

Supplement: Supplementary file 7 — Supplementary video 4 [file 44172_2023_159_MOESM7_ESM.gif]
