# Supplementary material for: A tunable acoustic absorber using reconfigurable dielectric elastomer actuated petals
Source: Commun Eng. 2024 Jan 10;3:11. doi: 10.1038/s44172-023-00159-z (PMC10955946; doi:10.1038/s44172-023-00159-z)
Supplement: Supplementary file 1 — Peer Review File [file 44172_2023_159_MOESM1_ESM.pdf]

nature portfolio

Peer Review File

A tunable acoustic absorber using reconfigurable dielectric elastomer actuated petals

Open Access This file is licensed under a Creative Commons Attribution 4.0 International License, which permits use, sharing, adaptation, distribution and reproduction in any medium or format, as long as you give appropriate credit to the original author(s) and the source, provide a link to the Creative Commons license, and indicate if changes were made. In the cases where the authors are anonymous, such as is the case for the reports of anonymous peer reviewers, author attribution should be to 'Anonymous Referee' followed by a clear attribution to the source work. The images or other third party material in this file are included in the article's Creative Commons license, unless indicated otherwise in a credit line to the material. If material is not included in the article's Creative Commons license and your intended use is not permitted by statutory regulation or exceeds the permitted use, you will need to obtain permission directly from the copyright holder. To view a copy of this license, visit http://creativecommons.org/licenses/by/4.0/.

Reviewers' comments:

Reviewer #1 (Remarks to the Author):

This paper seeks the inspiration from the deformations of flower petals to create a type of acoustic metasurface that can be reconfigured by a high-voltage activation. The effective open ratio of the acoustic metasurface can be altered to adapt to variations in the frequency of incident sound. I read this paper with interest because devising novel types of tunable acoustic metasurfaces is a fascinating research topic. I have a few remarks that I would like to share with the authors.

1. There are too many abbreviations in the entire paper, which can easily confuse the readers. I suggest the authors to reduce the use of abbreviations and further improve the readability of the paper.
2. Page 2, the authors mention that, these devices (micro-perforated dielectric elastomer actuators) can only survive for a few months as the stretched elastomer membrane with holes ruptures in a short period of time, making them impractical for real applications. Compared with these micro-perforated dielectric elastomer actuators, how long can the life of the proposed reconfigurable dielectric elastomer actuated petals be extended in this paper? It is suggested that the authors would provide some data on the number of rechargeable discharge cycles of the proposed reconfigurable dielectric elastomer actuated petals.
3. Each of the proposed reconfigurable dielectric elastomer actuated petals consists of a multi-layered dielectric elastomer actuator with one non-stretchable and flexible layer PET layer [see Figure 1(c-d)]. The multi-layered dielectric elastomer actuator consists of three soft VHB layers each of 250μm thickness. Why is it necessary to use three soft VHB layers (or three dielectric elastomer layers)? In this way, each layer needs to add electrical leads, resulting in a more complex structure. Hence, what would happen if only two layers or even one layer were used? Would it still be effective enough?
4. How are the error bars in Figure 3 calculated? Were multiple specimens tested or was one specimen measured multiple times?
5. Page 6, the proposed device has a response time of approximately 12 seconds to reach 90% of its final activated state and approximately a second to reach its flat deactivated state. It is suggested that the authors should analyze the reasons for different response times of the activated and deactivated states.
6. Page 6, numerical and experiment tests were done to select the optimized parameters for such a parallel-arranged design. What are the specific optimization methods? Please supplement this part of the content.
7. Page 9, the authors mention that, the absorption coefficient measured at normal incidence of sound shows the proposed absorber is versatile and can effectively attenuate sound and tune the absorption peaks despite any angle of incidence of the sound. Because the results of normal incidence in the experiments are not suitable for directly inferring the results of oblique incidence, it is recommended that the authors supplement the simulated or experimental results for the cases of oblique incidence.

Reviewer #2 (Remarks to the Author):

The manuscript entitled “Reconfigurable Dielectric Elastomer Actuated Petals for Tunable Acoustic Metasurface” presents a tunable acoustic absorber with micro-slotted dielectric elastomer bending

actuators (MSDEBA). The voltage activation can increase the open ratio of the MSDEBA and shit the working bands of the acoustic absorber to higher frequencies. The active control of acoustic absorbers can provide improved absorption tunability and capacity than passive acoustic absorbers, which is a promising research direction for the next generation of acoustic absorbers. Although the proposed acoustic absorbers in this manuscript have not been optimized, the presented design concept is fresh and intriguing and the results are convincing. Therefore, I would like to recommend accepting the manuscript for publication in Communications Engineering after revisions. My comments and questions are as follows,

1. Acoustic metasurfaces generally have thicknesses significantly smaller than the working wavelength. Therefore, if the ratio between structural thickness and the lowest working wavelength is lager than 1/10, I suggest the authors revise the description of acoustic meatsurface" as "acoustic absorbers".
2. The review of the research background of acoustic absorbers needs improvement. For instance, many previous studies have achieved broadband absorption with ventilation. To name a few,
1) Dong, Ruizhi, et al. "Ultrabroadband acoustic ventilation barriers via hybrid-functional metasurfaces." Physical Review Applied 15.2 (2021): 024044.
2) Kumar, Sanjay, and Heow Pueh Lee. "Labyrinthine acoustic metastructures enabling broadband sound absorption and ventilation." Applied Physics Letters 116.13 (2020).
3) Xiang, Xiao, et al. "Ultra-open ventilated metamaterial absorbers for sound-silencing applications in environment with free air flows." Extreme Mechanics Letters 39 (2020): 100786.

In addition, I also recommend the authors to properly enrich the Introduction part regarding the metamaterial-based acoustic absorbers because there are .many high-performance tunable or broadband absorbers presented in recent years are not discussed. Some most-recent reviews in the field of acoustic absorbers may facilitate it :
1) Huang, Sibo, et al. "Sound-Absorbing Materials." Physical Review Applied 20.1 (2023): 010501.
2) Yang, Min, and Ping Sheng. "Acoustic metamaterial absorbers: The path to commercialization." Applied Physics Letters 122.26 (2023).
3. This manuscript provides an impedance analysis of the presented structures. However, there are scant discussions based on the results of theoretical impedance models. Therefore, I suggest the authors add a brief discussion on how the increase of the open ratio of the MSDEBA affects the shift of working frequencies.
4. On page 8, it is stated that "As MSDEBA can be activated to open up and tune the absorption spectrum to a higher frequency range, obtaining a wider absorption bandwidth…." Why the opening up of the MSDEBA will lead to wider absorption bandwidth? The opening up of the MSDEBA leads to reduced acoustic resistance. And from this point of view, the absorption bandwidth should be decreased rather than increased. Please clarify this point. Besides, when comparing the working bandwidths, octave scales are usually used. For example, 400-800 Hz and 800-1600 Hz have the same bandwidth because they are both one octave.
5. In Figure 5G and 5H, the definition of the shift frequency is unclear. For instance, in Fig. 5G, it seems that the absorption peak of the red line is corresponding to the first peak of the black line lower than 1059 Hz if the mode variation is traced.

Reviewer #3 (Remarks to the Author):

In this manuscript, the Authors report on a novel acoustic metasurface with unable properties, thanks to the integration of dielectric elastomer actuators used to modify the geometry of the orifices that open the surface to back cavities of varying depth. The modification of the opening size is at the origin of the tenable behaviour of the metasurface. In this sense, the Authors offer a novel contribution to a currently very lively area of research in acoustics. However, the work would benefit of a substantial sharpening of the message about the function and the capabilities of this novel metasurface.

For one, the Authors come close to claiming that the described metasurface is bioinspired in its design and to do so, they refer to various works that treat some aspect of the interaction of plant related systems with sound waves. However, none of the described interactions is closely related with the described metasurface, other than for the fact that the bending actuators are compared to flower petals. It is the reviewer's opinion that Bioinspiration can indeed be claimed in the unimorph mechanisms described in the manuscript. More care should be given to citing work of other Authors that describe similar concepts (some examples given in the annotated manuscript)

The Authors offer results of measurements for a multiplicity of different metasurface configurations. While this is generally commendable and valuable for the interested Reader, the message could become clearer by limiting the number of configurations shown in the main body of the manuscript, moving a majority of them to the supplementary materials and creating a conclusions section in which a take-home message for the Reader is clearly formulated, especially with respect to the mechanisms at play in the absorption behaviour of the metasurface and to the way that the actuated orifices affect them.

09/10/2023

Subject: Response Letter to the Request of Revision for Submission COMMSENG-23-0179

Dear Reviewers,

We are very excited to learn that you have taken interest in our manuscript “COMMSENG-23-0179: Reconfigurable Dielectric Elastomer Actuated Petals for Tunable Acoustic Absorbers” and the reviewers’ comments are positive with some suggestions for revision. We have tried to address the reviewers’ concerns and comments to the best of our knowledge and capabilities.

The following are the replies to the reviewers’ comments in **blue fonts** and changes made in the revised manuscript and are highlighted as per your suggestions:

Reviewers’ comments

Reviewer #1 (Remarks to the Author):

This paper seeks the inspiration from the deformations of flower petals to create a type of acoustic metasurface that can be reconfigured by a high-voltage activation. The effective open ratio of the acoustic metasurface can be altered to adapt to variations in the frequency of incident sound. I read this paper with interest because devising novel types of tunable acoustic metasurfaces is a fascinating research topic. I have a few remarks that I would like to share with the authors.

1. There are too many abbreviations in the entire paper, which can easily confuse the readers. I suggest the authors to reduce the use of abbreviations and further improve the readability of the paper.

Reply: Thanks for the suggestion. As advised, we have reduced the number of abbreviations and added a list of abbreviations to guide the readers for the frequently used and known abbreviations in the field are added in (lines 38-45).

2. On Page 2, the authors mention that, these devices (micro-perforated dielectric elastomer actuators) can only survive for a few months as the stretched elastomer membrane with holes ruptures in a short period of time, making them impractical for real applications. Compared with these micro-perforated dielectric elastomer actuators, how long can the life of the proposed reconfigurable dielectric elastomer actuated petals be extended in this paper? It is suggested that the authors would provide some data on the number of rechargeable discharge cycles of the proposed reconfigurable dielectric elastomer actuated petals.

Reply: To further clarify, the micro-perforated dielectric elastomer actuators are made of stretched elastomer membranes with holes. We observed that over time the elastomer ruptures (within a few months due to the Creep of the viscoelastic

elastomer material). Taking this observation as a motivation, we proposed the device in this paper which is free of pre-tension and holes. In addition, the membranes are bonded to a stiff flexible PET membrane, hence, the device will not fail due to creep or mechanical rupture. This point has been clarified in lines 83-84, 112-114. Regarding the activation cycle of the reconfigurable dielectric elastomer actuated petals, we observed it can last 100s of activation cycles, but we could not evaluate further reliability within the scope of this paper. Thank you.

3. Each of the proposed reconfigurable dielectric elastomer actuated petals consists of a multi-layered dielectric elastomer actuator with one non-stretchable and flexible layer PET layer [see Figure 1(c-d)]. The multi-layered dielectric elastomer actuator consists of three soft VHB layers each of 250μm thickness. Why is it necessary to use three soft VHB layers (or three dielectric elastomer layers)? In this way, each layer needs to add electrical leads, resulting in a more complex structure. Hence, what would happen if only two layers or even one layer was used? Would it still be effective enough?

Reply: To answer your question, multilayered DEAs with thinner layers were introduced to reduce the applied voltage of the DEAs. If only one layer of dielectric elastomer of thickness T is used instead of 3 thinner layers with the same total thickness (i.e. T=3*t), the applied voltage will need to be approximately 3 times higher. To further clarify, DEA works based on Maxwell stress which is directly proportional to the applied electric field E=V/t (see equation 1). Hence, to obtain an equal amount of bending strain the electric field E must be the same as well. Now, if a single elastomer layer of thickness T=750μm is used instead of 3 layers of t=250μm, the applied voltage will be V_1 = E*T = E*3*t = 3*V, which is 3 times higher to V=E*t. In addition, to obtain the required voltage-activated bending, the total thickness (T=3*t) of the elastomer layers must be 750μm for the PET thickness of 25μm. The use of an elastomer layer with a lower total thickness will reduce the obtainable bending actuation (supported by Timoshenko beam theory). This point has been added in lines 132-133.

4. How are the error bars in Figure 3 calculated? Were multiple specimens tested or was one specimen measured multiple times?

Reply: Multiple specimens were tested to evaluate the error bars in Figure 3. It has been mentioned in the revised manuscript in line 200. Thank you.

5. Page 6, the proposed device has a response time of approximately 12 seconds to reach 90% of its final activated state and approximately a second to reach its flat deactivated state. It is suggested that the authors should analyze the reasons for the different response times of the activated and deactivated states.

Reply: Thanks for the suggestions. Indeed, the dielectric elastomers used for the MSDEBAs are well-known non-linear viscoelastic acrylic elastomer materials widely used to make dielectric elastomer actuators. The non-linear viscoelastic nature of these materials is well documented and modelled in the literature and it is known to be the main cause for the viscoelastic drift that increases the response time

during activation of DEAs. In addition, such viscoelastic elastomer shows significant hysteresis (strain vs electric field) which are known cause for variation in the response time during activation and deactivation cycles. This point has been clarified with the addition of appropriate literature citations in lines 207-211.

6. Page 6, numerical and experiment tests were done to select the optimized parameters for such a parallel-arranged design. What are the specific optimization methods? Please supplement this part of the content

Reply: Thanks for the comment. To clarify, in this study no specific design of experiment (DOE) protocol was followed as the focus was on the tunability of these absorbers. However, we studied the effect of depth of the parallel-arrange back-cavity design by changing the values of the cavity depths: D1 to D5 in over 40 combination cases through simulation and experimentally validated a few simulation cases (see Figure S1 in supplementary files). Among the simulated cases of the parallel-arrange design, the one showing a significant and broad absorption spectrum at a lower frequency was selected to showcase the tunable acoustic absorber concept.

To make this clear, we have modified the sentence in lines 228-232 to, "Due to constraints from acoustic measurement setup and commercial application requirements the overall dimension of the unit absorber was set to be 200mm x 200mm x 50mm. Numerical and experiment tests were done for over 40 cases with variations of D1 to D5 (i.e., depths of the parallel back-cavities) while keeping their widths equal and constant. Eventually, the parallel-arranged back-cavity depths D1 to D5 that gave optimal and broad absorption at lower frequency were selected."

7. On Page 9, the authors mention that, the absorption coefficient measured at normal incidence of sound shows the proposed absorber is versatile and can effectively attenuate sound and tune the absorption peaks despite any angle of incidence of the sound. Because the results of normal incidence in the experiments are not suitable for directly inferring the results of oblique incidence, it is recommended that the authors supplement the simulated or experimental results for the cases of oblique incidence.

Reply: Thanks for the comment. To clarify, we have presented two set of experimental acoustic characterizations in the manuscript, namely acoustic absorption, and transmission loss measurements. As shown in the figures of the measurement setups in Figure 6, the device is mounted in normal incidence (90°) to the source of sound in absorption measurement and in parallel incidence (180°) in the transmission loss measurements. Therefore, we want to express that the device might be usable in various sound incidence scenarios. However, at this point, as the measurement setup in oblique incidence is not available, we have modified this claim in the manuscript to avoid conflict (refer to lines 316-318).

Reviewer #2 (Remarks to the Author):

The manuscript entitled "Reconfigurable Dielectric Elastomer Actuated Petals for Tunable Acoustic Metasurface" presents a tunable acoustic absorber with micro-slotted dielectric elastomer bending actuators (MSDEBA). The voltage activation can increase the open ratio of the MSDEBA and shit the working bands of the acoustic absorber to higher frequencies. The active control of acoustic absorbers can provide improved absorption tunability and capacity than passive acoustic absorbers, which is a promising research direction for the next generation of acoustic absorbers. Although the proposed acoustic absorbers in this manuscript have not been optimized, the presented design concept is fresh and intriguing, and the results are convincing. Therefore, I would like to recommend accepting the manuscript for publication in Communications Engineering after revisions. My comments and questions are as follows,

1. Acoustic metasurfaces generally have thicknesses significantly smaller than the working wavelength. Therefore, if the ratio between structural thickness and the lowest working wavelength is larger than 1/10, I suggest the authors revise the description of acoustic metasurfaces" as "acoustic absorbers".

Reply: Thank you for the suggestions. As advised, we have revised the description of whole system to be "acoustic absorbers" in title and though out the manuscript as these device have a thickness of 5cm and working frequency range of 400-1600Hz.

2. The review of the research background of acoustic absorbers needs improvement. For instance, many previous studies have achieved broadband absorption with ventilation. To name a few,

i. Dong, Ruizhi, et al. "Ultrabroadband acoustic ventilation barriers via hybrid-functional metasurfaces." Physical Review Applied 15.2 (2021): 024044.
ii. Kumar, Sanjay, and Heow Pueh Lee. "Labyrinthine acoustic metastructures enabling broadband sound absorption and ventilation." Applied Physics Letters 116.13 (2020).
iii. Xiang, Xiao, et al. "Ultra-open ventilated metamaterial absorbers for sound-silencing applications in environment with free air flows." Extreme Mechanics Letters 39 (2020): 100786.

Reply: We appreciate the reviewer's suggestions. We have added a short discussion and cited the above articles in the introduction section of the revised manuscript in lines 63-64. Thank you.

In addition, I also recommend the authors to properly enrich the Introduction part regarding the metamaterial-based acoustic absorbers because there are many high-performance tunable or broadband absorbers presented in recent years are not discussed. Some most recent reviews in the field of acoustic absorbers may facilitate it:

i. Huang, Sibo, et al. "Sound-Absorbing Materials." Physical Review Applied 20.1 (2023): 010501.

ii. Yang, Min, and Ping Sheng. "Acoustic metamaterial absorbers: The path to commercialization." Applied Physics Letters 122.26 (2023.

Reply: As advised, we have discussed more recent literature on sound-absorbing materials in lines 58-60, 63-64 and 89-80. We have also cited the suggested articles in line 60 and a few more publications to enrich the introduction section (see line 80).

3. This manuscript provides an impedance analysis of the presented structures. However, there are scant discussions based on the results of theoretical impedance models. Therefore, I suggest the authors add a brief discussion on how the increase of the open ratio of the MSDEBA affects the shift of working frequencies.

Reply: Thanks for the comment. As advised to clarify the relationship of the open ratio to the absorption spectrum we have added the following statement in the manuscript (refer to lines 257-266): "Based on the electroacoustic model in **Error! Reference source not found.**.D, the addition of MSP to the MSDEBA layer increases the acoustic resistance as well as the reactance of the absorber. This is manifested by the occurrence of the resonant peaks at lower frequencies in the absorption spectrum and a slight reduction of the absorption coefficient at those resonant frequencies (refer to supplementary file's Figure S3.F). Meanwhile for the combined reconfigurable system, based on Equations (1,2 and 4) the projected open ratio ($\phi(V)$) = A_V/b^2 has a quartic increase with the increase in the external control voltage as shown in **Error! Reference source not found.**.E, which increases the overall porosity of the MSP/MSDEBA panel. As the open ratio $\phi(V)$ is increased, like any MPP or combination of MPPs[59-61], it reduces the acoustic reactance of the system following Equations 5 and 7. Consequently, the resonant absorption peaks are shifted to higher frequencies. This suggests, with a higher voltage causing a larger opening of the MSDEBA, a larger frequency shift will be achieved."

4. On page 8, it is stated that "As MSDEBA can be activated to open up and tune the absorption spectrum to a higher frequency range, obtaining a wider absorption bandwidth…." Why the opening up of the MSDEBA will lead to wider absorption bandwidth? The opening up of the MSDEBA leads to reduced acoustic resistance. And from this point of view, the absorption bandwidth should be decreased rather than increased. Please clarify this point. Besides, when comparing the working bandwidths, octave scales are usually used. For example, 400-800 Hz and 800-1600 Hz have the same bandwidth because they are both one octave.

Reply: We agree with the reviewer's comment regarding the relationship between acoustic resistance and absorption bandwidth; and have realized that our statement was not clear. We wanted to state, that MSDEBA can be activated to open up and tune the peak absorption spectrum to higher frequencies. Hence, this tunability allows the MSDEBA absorbers to actively target the noise within a wider frequency range compared to their passive counterpart. This point has been revised for clarity in lines 286-288. Thank you.

5. In Figures 5G and 5H, the definition of the shift frequency is unclear. For instance, in Fig. 5G, it seems that the absorption peak of the red line is corresponding to the first peak of the black line lower than 1059 Hz if the mode variation is traced.

Reply: As advised, we have tried to clarify the intended meaning of "shift in frequency". We meant to express that at the voltage-off state, the MSDEBA absorber has a peak absorption at a lower frequency; upon activation of the MSDEBA absorber, the peak absorption occurs at a higher frequency. We have revised the manuscript content to clearly express this intended meaning (see lines 292, 294, 300,301). Thank you.

Reviewer #3 (Remarks to the Author):

In this manuscript, the Authors report on a novel acoustic metasurface with tunable properties, thanks to the integration of dielectric elastomer actuators used to modify the geometry of the orifices that open the surface-to-back cavities of varying depth. The modification of the opening size is at the origin of the tenable behaviour of the metasurface. In this sense, the Authors offer a novel contribution to a currently very lively area of research in acoustics. However, the work would benefit of a substantial sharpening of the message about the function and the capabilities of this novel metasurface.

Reply: Thank you for the comments. As advised, we have revised and re-written the content for appropriate message delivery regarding the function and capabilities of the device. The changes are marked throughout the revised manuscript for ease of visualization.

For one, the Authors come close to claiming that the described metasurface is bioinspired in its design and to do so, they refer to various works that treat some aspect of the interaction of plant related systems with sound waves. However, none of the described interactions is closely related with the described metasurface, other than for the fact that the bending actuators are compared to flower petals. It is the reviewer's opinion that Bioinspiration can indeed be claimed in the unimorph mechanisms described in the manuscript. More care should be given to citing work of other Authors that describe similar concepts (some examples given in the annotated manuscript)

Reply: As advised by the reviewer, we have revised and removed few contents in the introduction section that was not very closely related to our system and revised content for better understanding and relevance (see lines 98-101). (Please note that paragraph on flower blooming when stimulated by various phenomenon has been removed). In addition, we have cited more work on the DEA-based unimorph mechanism to facilitate a better understanding of the mechanism for the readers (see lines 104 and 107). Thank you.

The Authors offer results of measurements for a multiplicity of different metasurface configurations. While this is generally commendable and valuable for the interested Reader, the message could become clearer by limiting the number of configurations shown in the main body of the manuscript, moving a majority of them to the supplementary materials and creating a conclusions section in which a take-home message for the Reader is formulated, especially with respect to the mechanisms at play in the absorption behaviour of the metasurface and to the way that the actuated orifices affect them.

Reply: We appreciate the reviewer’s comment and as advised we have moved two basic configurations of the acoustic absorber that was presented in Figure 5 to supplementary files (refer to Figure S3 in supplementary) and focused our discussion on MSDEBA based absorbers to better deliver the intended message (see lines 268-275). Thank you.

We appreciate your effort and time to go through the manuscript and give us such positive and constructive feedback. We have tried to address all your comments and are looking forward to your positive response. Thank you.

Best regards,

Milan Shrestha (PhD)

Senior Research Fellow,

Nanyang Technological University, Singapore

Reviewers' comments:

Reviewer #1 (Remarks to the Author):

The authors have improved substantially the manuscript and in my opinion, it is now in a publishable form.

Reviewer #2 (Remarks to the Author):

The authors have addressed all of my comments. Therefore, I would like to recommend accepting this manuscript for publication in Communications Engineering.

Reviewer #3 (Remarks to the Author):

A comparison of revised manuscript with the annotated manuscript shared by the reviewer with the Authors indicates that most if not all the annotations made in the manuscript and intended as a guidance for the Authors were ignored and no rebuttal was made to any of them. However, the reviewer still maintains that there is a merit to the annotations. Possibly the Authors overlooked them.

Please revise the manuscript taking the comments in consideration or address them in the rebuttal with a comment on why there are not regarded.

29/10/2023

Subject: Response Letter to the Request of Revision for Submission COMMSENG-23-0179 with **additional comments from Reviewer 3 in the annotated manuscript**.

Dear Reviewers,

We are very excited to learn that you have taken interest in our manuscript "COMMSENG-23-0179: Reconfigurable Dielectric Elastomer Actuated Petals for Tunable Acoustic Absorbers" and the reviewers' comments are positive with some suggestions for revision. **In the previous revision, due to some error, we did not receive the annotated manuscript from Reviewer 3. However, now we have received the annotated comments in the manuscript, and we have tried to address all the reviewers' concerns and comments to the best of our knowledge and capabilities.**

The following are the replies to the reviewers' comments in **blue fonts** and changes made in the revised manuscript are highlighted as per your suggestions. (Please note that reviewer 3's comments in the annotated manuscript have been addressed here as well:

Reviewers' comments

Reviewer #1 (Remarks to the Author):

This paper seeks the inspiration from the deformations of flower petals to create a type of acoustic metasurface that can be reconfigured by a high-voltage activation. The effective open ratio of the acoustic metasurface can be altered to adapt to variations in the frequency of incident sound. I read this paper with interest because devising novel types of tunable acoustic metasurfaces is a fascinating research topic. I have a few remarks that I would like to share with the authors.

1. There are too many abbreviations in the entire paper, which can easily confuse the readers. I suggest the authors to reduce the use of abbreviations and further improve the readability of the paper.

Reply: Thanks for the suggestion. As advised, we have reduced the number of abbreviations and added a list of abbreviations to guide the readers for the frequently used and known abbreviations in the field are added in (lines 39-46).

2. On Page 2, the authors mention that, these devices (micro-perforated dielectric elastomer actuators) can only survive for a few months as the stretched elastomer membrane with holes ruptures in a short period of time, making them impractical for real applications. Compared with these micro-perforated dielectric elastomer actuators, how long can the life of the proposed reconfigurable dielectric elastomer actuated petals be extended in this paper? It is suggested that the authors would

provide some data on the number of rechargeable discharge cycles of the proposed reconfigurable dielectric elastomer actuated petals.

Reply: To further clarify, the micro-perforated dielectric elastomer actuators are made of stretched elastomer membranes with holes. We observed that over time the elastomer ruptures (within a few months due to the Creep of the viscoelastic elastomer material). Taking this observation as a motivation, we proposed the device in this paper which is free of pre-tension and holes. In addition, the membranes are bonded to a stiff flexible PET membrane, hence, the device will not fail due to creep or mechanical rupture. This point has been clarified in lines 84-85, 117-118. Regarding the activation cycle of the reconfigurable dielectric elastomer actuated petals, we observed it can last 100s of activation cycles, but we could not evaluate further reliability within the scope of this paper. Thank you.

3. Each of the proposed reconfigurable dielectric elastomer actuated petals consists of a multi-layered dielectric elastomer actuator with one non-stretchable and flexible layer PET layer [see Figure 1(c-d)]. The multi-layered dielectric elastomer actuator consists of three soft VHB layers each of 250 μm thickness. Why is it necessary to use three soft VHB layers (or three dielectric elastomer layers)? In this way, each layer needs to add electrical leads, resulting in a more complex structure. Hence, what would happen if only two layers or even one layer was used? Would it still be effective enough?

Reply: To answer your question, multilayered DEAs with thinner layers were introduced to reduce the applied voltage of the DEAs. If only one layer of dielectric elastomer of thickness T is used instead of 3 thinner layers with the same total thickness (i.e. T=3*t), the applied voltage will need to be approximately 3 times higher. To further clarify, DEA works based on Maxwell stress which is directly proportional to the applied electric field E=V/t (see equation 1). Hence, to obtain an equal amount of bending strain the electric field E must be the same as well. Now, if a single elastomer layer of thickness T=750μm is used instead of 3 layers of t=250μm, the applied voltage will be V_1 = E*T = E*3*t = 3*V, which is 3 times higher to V=E*t. In addition, to obtain the required voltage-activated bending, the total thickness (T=3*t) of the elastomer layers must be 750μm for the PET thickness of 25μm. The use of an elastomer layer with a lower total thickness will reduce the obtainable bending actuation (supported by Timoshenko beam theory). This point has been added in lines 138-139.

4. How are the error bars in Figure 3 calculated? Were multiple specimens tested or was one specimen measured multiple times?

Reply: Multiple specimens were tested to evaluate the error bars in Figure 3. It has been mentioned in the revised manuscript in line 206. Thank you.

5. Page 6, the proposed device has a response time of approximately 12 seconds to reach 90% of its final activated state and approximately a second to reach its flat deactivated state. It is suggested that the authors should analyze the reasons for the different response times of the activated and deactivated states.

Reply: Thanks for the suggestions. Indeed, the dielectric elastomers used for the MSDEBAs are well-known non-linear viscoelastic acrylic elastomer materials widely used to make dielectric elastomer actuators. The non-linear viscoelastic nature of these materials is well documented and modelled in the literature and it is known to be the main cause for the viscoelastic drift that increases the response time during activation of DEAs. In addition, such viscoelastic elastomer shows significant hysteresis (strain vs electric field) which are known cause for variation in the response time during activation and deactivation cycles. This point has been clarified with the addition of appropriate literature citations in lines 214-217.

6. Page 6, numerical and experiment tests were done to select the optimized parameters for such a parallel-arranged design. What are the specific optimization methods? Please supplement this part of the content

Reply: Thanks for the comment. To clarify, in this study no specific design of experiment (DOE) protocol was followed as the focus was on the tunability of these absorbers. However, we studied the effect of depth of the parallel-arrange back-cavity design by changing the values of the cavity depths: D1 to D5 in over 40 combination cases through simulation and experimentally validated a few simulation cases (see Figure S1 in supplementary files). Among the simulated cases of the parallel-arrange design, the one showing a significant and broad absorption spectrum at a lower frequency was selected to showcase the tunable acoustic absorber concept. To make this clear, we have modified the sentence in lines 234-238 to, "Due to constraints from acoustic measurement setup and commercial application requirements the overall dimension of the unit absorber was set to be 200mm x 200mm x 50mm. Numerical and experiment tests were done for over 40 cases with variations of D1 to D5 (i.e., depths of the parallel back-cavities) while keeping their widths equal and constant. Eventually, the parallel-arranged back-cavity depths D1 to D5 that gave optimal and broad absorption at lower frequency were selected."

7. On Page 9, the authors mention that, the absorption coefficient measured at normal incidence of sound shows the proposed absorber is versatile and can effectively attenuate sound and tune the absorption peaks despite any angle of incidence of the sound. Because the results of normal incidence in the experiments are not suitable for directly inferring the results of oblique incidence, it is recommended that the authors supplement the simulated or experimental results for the cases of oblique incidence.

Reply: Thanks for the comment. To clarify, we have presented two set of experimental acoustic characterizations in the manuscript, namely acoustic absorption, and transmission loss measurements. As shown in the figures of the measurement setups in Figure 6, the device is mounted in normal incidence (90°) to the source of sound in absorption measurement and in parallel incidence (180°) in the transmission loss measurements. Therefore, we want to express that the device might be usable in various sound incidence scenarios. However, at this point, as the measurement setup

in oblique incidence is not available, we have modified this claim in the manuscript to avoid conflict (refer to lines 322-324).

Reviewer #2 (Remarks to the Author):

The manuscript entitled "Reconfigurable Dielectric Elastomer Actuated Petals for Tunable Acoustic Metasurface" presents a tunable acoustic absorber with micro-slotted dielectric elastomer bending actuators (MSDEBA). The voltage activation can increase the open ratio of the MSDEBA and shit the working bands of the acoustic absorber to higher frequencies. The active control of acoustic absorbers can provide improved absorption tunability and capacity than passive acoustic absorbers, which is a promising research direction for the next generation of acoustic absorbers. Although the proposed acoustic absorbers in this manuscript have not been optimized, the presented design concept is fresh and intriguing, and the results are convincing. Therefore, I would like to recommend accepting the manuscript for publication in Communications Engineering after revisions. My comments and questions are as follows,

1. Acoustic metasurfaces generally have thicknesses significantly smaller than the working wavelength. Therefore, if the ratio between structural thickness and the lowest working wavelength is larger than 1/10, I suggest the authors revise the description of acoustic metasurfaces" as "acoustic absorbers".

Reply: Thank you for the suggestions. As advised, we have revised the description of whole system to be "acoustic absorbers" in title and though out the manuscript as these device have a thickness of 5cm and working frequency range of 400-1600Hz.

2. The review of the research background of acoustic absorbers needs improvement. For instance, many previous studies have achieved broadband absorption with ventilation. To name a few,

i. Dong, Ruizhi, et al. "Ultrabroadband acoustic ventilation barriers via hybrid-functional metasurfaces." Physical Review Applied 15.2 (2021): 024044.
ii. Kumar, Sanjay, and Heow Pueh Lee. "Labyrinthine acoustic metastructures enabling broadband sound absorption and ventilation." Applied Physics Letters 116.13 (2020).
iii. Xiang, Xiao, et al. "Ultra-open ventilated metamaterial absorbers for sound-silencing applications in environment with free air flows." Extreme Mechanics Letters 39 (2020): 100786.

Reply: We appreciate the reviewer's suggestions. We have added a short discussion and cited the above articles in the introduction section of the revised manuscript in lines 64-65. Thank you.

In addition, I also recommend the authors to properly enrich the Introduction part regarding the metamaterial-based acoustic absorbers because there are many high-performance tunable or broadband absorbers presented in recent years are not discussed. Some most recent reviews in the field of acoustic absorbers may facilitate it:

i. Huang, Sibo, et al. "Sound-Absorbing Materials." Physical Review Applied 20.1 (2023): 010501.
ii. Yang, Min, and Ping Sheng. "Acoustic metamaterial absorbers: The path to commercialization." Applied Physics Letters 122.26 (2023.

Reply: As advised, we have discussed more recent literature on sound-absorbing materials in lines 59-61, 64-65, and 80-81. We have also cited the suggested articles in line 60 and a few more publications to enrich the introduction section (see line 80).

3. This manuscript provides an impedance analysis of the presented structures. However, there are scant discussions based on the results of theoretical impedance models. Therefore, I suggest the authors add a brief discussion on how the increase of the open ratio of the MSDEBA affects the shift of working frequencies.

Reply: Thanks for the comment. As advised to clarify the relationship of the open ratio to the absorption spectrum we have added the following statement in the manuscript (refer to lines 263-272): "Based on the electroacoustic model in Figure 4.D, the addition of MSP to the MSDEBA layer increases the acoustic resistance as well as the reactance of the absorber. This is manifested by the occurrence of the resonant peaks at lower frequencies in the absorption spectrum and a slight reduction of the absorption coefficient at those resonant frequencies (refer to supplementary file' s Figure S3.F). Meanwhile for the combined reconfigurable system, based on Equations (1,2 and 4) the projected open ratio ($\phi(V)$) = A_V/b^2 has a quartic increase with the increase in the external control voltage as shown in Figure S3.E, which increases the overall porosity of the MSP/MSDEBA panel. As the open ratio $\phi(V)$ is increased, like any MPP or combination of MPPs[1-3], it reduces the acoustic reactance of the system following Equations 5 and 7. Consequently, the resonant absorption peaks are shifted to higher frequencies. This suggests, with a higher voltage causing a larger opening of the MSDEBA, a larger frequency shift will be achieved."

4. On page 8, it is stated that "As MSDEBA can be activated to open up and tune the absorption spectrum to a higher frequency range, obtaining a wider absorption bandwidth…." Why the opening up of the MSDEBA will lead to wider absorption bandwidth? The opening up of the MSDEBA leads to reduced acoustic resistance. And from this point of view, the absorption bandwidth should be decreased rather than increased. Please clarify this point. Besides, when comparing the working bandwidths, octave scales are usually used. For example, 400-800 Hz and 800-1600 Hz have the same bandwidth because they are both one octave.

Reply: We agree with the reviewer' s comment regarding the relationship between acoustic resistance and absorption bandwidth; and have realized that our statement was not clear. We wanted to state, that MSDEBA can be activated to open up and tune

the peak absorption spectrum to higher frequencies. Hence, this tunability allows the MSDEBA absorbers to actively target the noise within a wider frequency range compared to their passive counterpart. This point has been revised for clarity in lines 292-296. Thank you.

5. In Figures 5G and 5H, the definition of the shift frequency is unclear. For instance, in Fig. 5G, it seems that the absorption peak of the red line is corresponding to the first peak of the black line lower than 1059 Hz if the mode variation is traced.

Reply: As advised, we have tried to clarify the intended meaning of "shift in frequency". We meant to express that at the voltage-off state, the MSDEBA absorber has a peak absorption at a lower frequency; upon activation of the MSDEBA absorber, the peak absorption occurs at a higher frequency. We have revised the manuscript content to clearly express this intended meaning (see lines 298, 299, 300, 306, 307). Thank you.

Reviewer #3 (Remarks to the Author):

In this manuscript, the Authors report on a novel acoustic metasurface with tunable properties, thanks to the integration of dielectric elastomer actuators used to modify the geometry of the orifices that open the surface-to-back cavities of varying depth. The modification of the opening size is at the origin of the tenable behaviour of the metasurface. In this sense, the Authors offer a novel contribution to a currently very lively area of research in acoustics. However, the work would benefit of a substantial sharpening of the message about the function and the capabilities of this novel metasurface.

Reply: Thank you for the comments. As advised, we have revised and re-written the content for appropriate message delivery regarding the function and capabilities of the device. The changes are marked throughout the revised manuscript for ease of visualization.

For one, the Authors come close to claiming that the described metasurface is bioinspired in its design and to do so, they refer to various works that treat some aspect of the interaction of plant related systems with sound waves. However, none of the described interactions is closely related with the described metasurface, other than for the fact that the bending actuators are compared to flower petals. It is the reviewer's opinion that Bioinspiration can indeed be claimed in the unimorph mechanisms described in the manuscript. More care should be given to citing work of other Authors that describe similar concepts (some examples given in the annotated manuscript)

Reply: As advised by the reviewer, we have revised and removed a few contents in the introduction section that were not very closely related to our system and revised content for better understanding and relevance (see lines 98-105). (Please

note that the paragraph on flower blooming when stimulated by various phenomena has been removed). In addition, we have cited more work on the DEA-based unimorph mechanism to facilitate a better understanding of the mechanism for the readers (see lines 109 and 111). Thank you.

The Authors offer results of measurements for a multiplicity of different metasurface configurations. While this is generally commendable and valuable for the interested Reader, the message could become clearer by limiting the number of configurations shown in the main body of the manuscript, moving a majority of them to the supplementary materials and creating a conclusions section in which a take-home message for the Reader is formulated, especially with respect to the mechanisms at play in the absorption behaviour of the metasurface and to the way that the actuated orifices affect them.

Reply: We appreciate the reviewer's comment and as advised we have moved two basic configurations of the acoustic absorber that were presented in Figure 5 to supplementary files (refer to Figure S3 in supplementary) and focused our discussion on MSDEBA-based absorbers to better deliver the intended message (see lines 268-275). Thank you.

Reviewer 3's Comments in Annotated Manuscript:

1. Page 1: DEA unimorphs: Shian, S., Bertoldi, K., & Clarke, D. R. (2015). Dielectric elastomer based "grippers" for soft robotics. Advanced Materials, 27(43), 6814-6819.

Reply: As suggested, we have used the term DEA unimorphs as a generic name (lines 17, 19, 20).

2. Page 1: Variations in frequency?

Reply: To make it clear, we have used the following phrase instead, "variation in the frequency of dominant sound" (line 21). Thank you.

3. Page 1: Check for effect of number of layers on performance

Reply: A smaller no. of layers will give lesser bending of the DEA unimorph at the same voltage activations. However, using too many no. of layers will stiffen the DEA unimorph and reduce the bending. This study has been shown by other papers and is not within the scope of this article. We have added citations of these papers here (see lines 109 and 111). Thank you.

4. Page 1: the term transmission loss appears somewhat inaccurate to the reviewer, as it implies the transmission of waves across the device. The reviewer proposes the use of the term 'attenuation performance' as discussed, for example in: Kim, D. Y., & Ih, J. G. (2020). Wideband reduction of in-duct noise using acoustic metamaterial with serially connected resonators made with MPP and cavities. Applied Physics Letters, 116(25)

Reply: As advised, we have used the term "attenuation performance" as a generic phrase in the abstract. However, sound transmission loss is the technical phrase accepted widely to denote the reduction of sound intensity by absorbers measured with the setup used for this study. For example, Moore, James A., and Richard H. Lyon. "Sound transmission loss characteristics of sandwich panel constructions." The Journal of the Acoustical Society of America 89.2 (1991): 777-791. Therefore, it has been used for the technical discussions. Thank you.

5. Page 2: This seems to be the description of a Helmholtz resonator. Also, in view of the work discussed later, consider rainbow systems: Jiménez, N., Romero-García, V., Pagneux, V., & Groby, J. P. (2017). Rainbow-trapping absorbers: Broadband, perfect and asymmetric sound absorption by subwavelength panels for transmission problems. Scientific reports, 7(1), 13595.

Reply: Yes, when simplified to a very basic form, these absorbers are based on Helmholtz resonators. Therefore, the recommended publication is relevant to our parallel arranged varying back-cavity volume and we have cited this publication in line 223. Thank you.

6. Page 2: Interesting but somewhat beside the point, as the function of the proposed absorber is very different.

Reply: Yes, we agree with the reviewer' s comment, and we have removed the paragraph and revised part is in lines 98-105. Thank you.

7. Page 2: Indeed, the bio-inspiration argument is valid, however the examples cited indicate a design based on a biomimicry approach rather than a true bio-inspiration, as described in: Vincent, J. F. (2008). Biomimetic materials. Journal of Materials Research, 23(12), 3140-3147.

Reply: Yes, indeed this work tried to take into account that leaves and flowers are natural sound absorbers and we tried to mimic the flowers' blooming motion to make a tunable absorber. This point has been further clarified in lines (98-105)

8. Page 2: It would be interesting to refer to works on nastic motion and turgid structures, as these are the mechanisms often encountered in such cases. It could be a topic to mention in the introductory section.

Reply: As advised, we have added a short discussion on nastic motion and turgid structures with citations of some papers in the introduction section. (See lines 98-100) Thank you.

9. Page 3: it could be misinterpreted as the growth of the plant over time, as it matures.

Reply: The movement of petals in flowers could be due to both expansion of the cells in a short period of time or growth in the number of cells over time. We have clarified this point in line 128.

10. Page 3: If you mention this point, please be more specific: what overlaps what and how avoiding overlapping is beneficial for ease of fabrication?

Reply: While designing the dielectric elastomer petals, unlike real petals of most flowers, we avoided overlapping of the dielectric elastomer petals so that we could create individual DE petals by 2D laser cutting (see Figure 6.A). This point has been clarified in lines 134-135.

11. Page 3: many biological (plant) structures have a similar behavior. For example, pine cones inspired the work of many groups, see for example: Reyssat, E., & Mahadevan, L. (2009). Hygromorphs: from pine cones to biomimetic bilayers. Journal of the Royal Society Interface, 6(39), 951-957, in a truly biomimetic sense. Or more recently: Erb, R. M., Sander, J. S., Grisch, R., & Studart, A. R. (2013). Self-shaping composites with programmable bioinspired microstructures. Nature communications, 4(1), 1712.

Reply: Thanks for the suggestion, we have added this point and cited these publications as well in lines 135-136.

12. Page 3: what is the rationale for a multilayered VHB actuator? Why 3 layers? The further away from the VHB-PET interface, the larger the in-plane strain has to be in order to obtain high curvature. Are the different layers actuated at different voltage levels?

Reply: To answer your question, multilayered DEAs with thinner layers were introduced to reduce the applied voltage of the DEAs. If only one layer of dielectric elastomer of thickness T is used instead of 3 thinner layers with the same total thickness (i.e. T=3*t), the applied voltage will need to be approximately 3 times higher. To further clarify, DEA works based on Maxwell stress which is directly proportional to the applied electric field E=V/t (see equation 1). Hence, to obtain an equal amount of bending strain the electric field E must be the same as well. Now, if a single elastomer layer of thickness T=750μm is used instead of 3 layers of t=250μm, the applied voltage will be V1 = E*T = E*3*t = 3*V, which is 3 times higher to V=E*t. Also, Yes we agree that further away from the VHB-PET interface, the larger the in-plane strain has to be in order to obtain high curvature, however to obtain the required voltage-activated bending, the total thickness (T=3*t) of the elastomer layers must be 750μm for the PET thickness of 25μm. The use of an elastomer layer with a lower total thickness will reduce the obtainable bending actuation (supported by Timoshenko beam theory). However, to simplify the actuating high-voltage setup and the device design all DEA layers were actuated with the same voltage. This point has been added in lines 138-139.

13. Page 3: Please, add a scale bar to the image, as -at least with the reviewer- the photograph elicits the impression that the object is 'building size'. Refer to above mentioned work on rainbow absorbers, as the structure is very similar.

Reply: As advised we have added a scale bar to the photo in Figure 1(f).

14. Page 4: the MSDBA is per se not an absorber but an element that controls the size of the neck of a Helmholtz resonator

Reply: As suggested, the word absorber has been removed in line 155.

15. Page 4: However, the actual opening is given by the superposition of the microslits and the projected open area. In this context, the Authors have not (yet) explained the role of the MSP.

Reply: In this sentence, the projected open area is meant only for the MSDEBA layer. To avoid confusion this has been added in line 187. The role of MSP has been explained in lines 263-266 as "addition of MSP to the MSDEBA layer increases the acoustic resistance as well as the reactance of the absorber. This is manifested by the occurrence of the resonant peaks at lower frequencies in the absorption spectrum and a slight reduction of the absorption coefficient at those resonant frequencies (refer to supplementary file' s Figure S3.F).

16. Page 5: this sentence is not complete

Reply: Thanks for pointing it out. This was a typo and has been corrected in line 190.

17. Page 5: It should be noted that the absorber, as shown in this image, appears to be substantially less transparent (and with more of a yellow tinge) than in the photograph shown in figure 1. VHB is indeed quite yellow. Please, comment

Reply: We agree that the view here appears slightly yellow and hazy. However, the yellowing in this picture is vastly due to the difference in background, lighting, and magnification. Here we choose this background so that the micro-slots and the opening of the MSDEBA become distinguishable for the ease of presentation.

18. Page 6: how does this compare to the data in: Di Lillo, L., Schmidt, A., Carnelli, D. A., Ermanni, P., Kovacs, G., Mazza, E., & Bergamini, A. (2012). Measurement of insulating and dielectric properties of acrylic elastomer membranes at high electric fields. Journal of Applied Physics, 111(2

Reply: Thanks for the comment. We studied this publication and found that the leakage current follows a similar trend with an increment in applied voltage or electric field as expected. However, the dielectric elastomer material, electrode material, pre-stretch ratio, thickness, total active area and number of DE layers used in this publication are different compared to our work. Therefore, we feel it is not very useful for the audience to make a direct comparison of the absolute values of the leakage current between these studies.

19. Page 7: this should correspond to the side length of the square projected opening of the MSDEBA. Is this correct? A certain discreteness should be observed, as the contribution of individual slits depends on where they fall relative to the projected opening. Is this the case?

Reply: This is not the case, l_l is simply the length of the micro-slots in the MSP (line 255). The reason is if you refer to the electro-acoustic model in Figure 4.D, here impedance of each component of the absorber, namely the Impedance of MSDEBA (Z_{MSDEBA}), Impedance of MSP (Z_{MSP}), and Impedance of back-cavity (Z_D) solely depend on their own geometrical/material properties and does not depend on each other. And final acoustic property of the system depends solely upon the total impedance of the system given by Equation 5.

20. Page 7: Wealth of results shown in this section and a difficult to discern systematic to them, makes it difficult for the Reader to distill a take home message. It would help to chose one (or a smaller selection of) setup(s) to sharpen the message and move other results to the supplementary materials. More care should be given to offering plausible explanations for the observed behaviors

Reply: We appreciate the reviewer's comment and as advised we have moved two basic configurations of the acoustic absorber that were previously presented in Figure 5 to supplementary files (refer to Figure S3 in supplementary) and focused our discussion on MSDEBA-based absorbers to better deliver the intended message (see lines 274-281). Thank you.

21. Page 8: A MSP + Normal back cavity would also be an interesting comparison.

Reply: This comparison is shown in supplementary files in Figure S1. Thank you.

22. Page 8: are all MSDEBA rows (for different depths of the back cavity activated with the same voltage? Could frequency-selective absorption be achieved, by selectively activating the MSDEBAs corresponding to a different depth.

Reply: Please note that the whole 5x5 array is made with a layer-by-layer approach in a single 200mm by 200mm MSDEBA layer. Therefore, all petals of MSDEBA in this study are activated with the same voltage. Yes, frequency-selective absorption could also be achieved by selectively activating the MSDEBAs corresponding to a different depth as well. However, this more complicated approach of fabrication and activation is not explored as a part of this study and could be a topic for future study. Thank you.

23. Page 10: developed in-house? commercial (if so, please specify). What is the minimum displacement that can be tracked? How is the tip marked so that it can be tracked?

Reply: Tracker is an open-source software freely available for use. The minimum distance that can be tracked depends on the resolution of the video. Therefore, for our case minimum displacement that can be tracked is about 0.18mm. The MSDEBA tip is marked by a black dot which is tracked using the software.

24. Page 10: At 1600Hz an acoustic wave in air has a length of approximately 20 cm (typical laboratory conditions). This could lead the the formation of standing waves across the impedance tube used for these experiments with consequent

inaccuracies (see, for example: Spillere, A., Cordioli, J. A., & Bodén, H. (2017). On the effect of boundary conditions on impedance eduction results. In 23rd AIAA/CEAS Aeroacoustics Conference (p. 3185).) Please, comment on this point.

Reply: We agree with the reviewer' s comment. However, please note that the impedance tube shown in Figure 6(b) with 200mm x 200mm cross-section is used for the absorption coefficient measurement; the cut-off frequency of this impedance tube is 850Hz, thus we plotted the results of the absorption coefficient of the sample in the frequency range from 200Hz to 850Hz on Figure 5(H). The results in Figure 5(E) and (F) are obtained using the transmission loss measurement setup which uses another acoustic duct shown in Figure 6(c). This acoustic duct has a cross-section of 200mm x 40mm and the main cut-off frequency of this duct is higher than 2000Hz (note: the wider dimension is 4 times more than the smaller dimensions and the results have been validated). This is the reason; we only plotted the transmission loss data in the frequency range from 400Hz to 1600Hz. Thank you.

We appreciate your effort and time to go through the manuscript and give us such positive and constructive feedback. We have tried to address all your comments and are looking forward to your positive response. Thank you.

Best regards,

Milan Shrestha (PhD)

Senior Research Fellow,

Nanyang Technological University, Singapore
